# The F-box protein MIO1/SLB1 regulates organ size and leaf movement in *Medicago truncatula*

**DOI:** 10.1093/jxb/erab033

**Published:** 2021-01-28

**Authors:** Shaoli Zhou, Tianquan Yang, Yawen Mao, Ye Liu, Shiqi Guo, Ruoruo Wang, Genwang Fangyue, Liangliang He, Baolin Zhao, Quanzi Bai, Youhan Li, Xiaojia Zhang, Dongfa Wang, Chaoqun Wang, Qing Wu, Yuanfan Yang, Yu Liu, Million Tadege, Jianghua Chen

**Affiliations:** 1 CAS Key Laboratory of Tropical Plant Resources and Sustainable Use, CAS Center for Excellence for Molecular Plant Sciences, Xishuangbanna Tropical Botanical Garden, Chinese Academy of Sciences, Kunming, Yunnan, China; 2 University of Chinese Academy of Sciences, Beijing, China; 3 Germplasm Bank of Wild Species, Kunming Institute of Botany, Chinese Academy of Sciences, Kunming, China; 4 School of Life Sciences, University of Science and Technology of China, Hefei, China; 5 School of Ecology and Environmental Sciences, Yunnan University, Kunming, China; 6 Department of Plant and Soil Sciences, Institute for Agricultural Biosciences, Oklahoma State University, 3210 Sam Noble Parkway, Ardmore, OK, USA; 7 University of Edinburgh, UK

**Keywords:** *BIG SEEDS1* (*BS1*), F-box protein, MINI ORGAN1 (MIO1)/SMALL LEAF AND BUSHY1 (SLB1), SCF E3 ligase, proteasome-mediated degradation, organ size, pulvinus

## Abstract

The size of leaf and seed organs, determined by the interplay of cell proliferation and expansion, is closely related to the final yield and quality of forage and crops. Yet the cellular and molecular mechanisms underlying organ size modulation remain poorly understood, especially in legumes. Here, *MINI ORGAN1* (*MIO1*), which encodes an F-box protein SMALL LEAF AND BUSHY1 (SLB1) recently reported to control lateral branching in *Medicago truncatula*, was identified as a key regulator of organ size. We show that loss-of-function of *MIO1/SLB1* severely reduced organ size. Conversely, plants overexpressing *MIO1*/*SLB1* had enlarged organs. Cellular analysis revealed that MIO1/SLB1 controlled organ size mainly by modulating primary cell proliferation during the early stages of leaf development. Biochemical analysis revealed that MIO1/SLB1 could form part of SKP1/Cullin/F-box (SCF) E3 ubiquitin ligase complex, to target BIG SEEDS1 (BS1), a repressor of primary cell division, for degradation. Interestingly, we found that MIO1/SLB1 also played a key role in pulvinus development and leaf movement by modulating cell proliferation of the pulvinus as leaves developed. Our study not only demonstrates a conserved role of MIO1/SLB1 in the control of organ size in legumes, but also sheds light on the novel function of MIO1/SLB1 in leaf movement.

## Teaser

The F-box protein MIO1/SLB1 regulates lateral organ size and pulvinus development by forming part of an SCF E3 ubiquitin ligase to modulate stability of BIG SEEDS1 in *Medicago truncatula*.

## Introduction

Organ size in plants, which is determined by their genetic make-up and environmental conditions, is one of the most important parameters for their adaptation and survival (Czesnick and Lenhard, 2015). In agriculture, the size of plant organs directly influences the final yield and quality of crops (Graham and Vance, 2003). In particular, legumes provide the most significant sources of plant-based protein for consumption by humans and animals. So, improving the production of legume crops and forage by increasing organ size is one promising way to meet the protein requirements of the rise in global population. However, the cellular and molecular mechanisms underlying the determination of organ size in plants remain largely unclear, especially in legume species.

In plants, organ size is controlled by a complex interplay of cell proliferation and expansion (Gonzalez *et al.*, 2012; Hepworth and Lenhard, 2014; Czesnick and Lenhard, 2015). The leaf has served as an ideal system to understand the plasticity of organ size in a developmental context. For Arabidopsis, leaf growth is under spatiotemporal control at five overlapping and interconnecting phases: initiation phase, when the founder cells are recruited to form the oldest incipient leaf primordium (P0) in the peripheral zone (PZ) of the shoot apical meristem (SAM; Sluis and Hake, 2015; Du *et al.*, 2018); general cell division phase, when cell proliferation occurs throughout the entire primordium during primary morphogenesis (Gonzalez *et al.*, 2012; Du *et al.*, 2018); transition phase, when cell division ceases in a basipetal manner (from the tip to the base of leaf primodium; Andriankaja *et al.*, 2012); cell expansion phase, when the cell size becomes enlarged, which generally coincides with endoreduplication, and remodeling of the cell wall and vacuole (Cosgrove, 2005; Cookson *et al*, 2006); and finally, meristemoid division phase, when the dispersed meristematic cells (DMC) undergo asymmetric division to form stomatal guard and stomatal-lineage ground cells (Donnelly *et al.*, 1999; White, 2006; Pillitteri and Torii, 2012). The final leaf size would thus be coordinated by the number of cells of the primodium, the rate and duration of general cell division, as well as cell expansion and meristemoid division (Gonzalez *et al.*, 2012). The disturbance of genetic pathways that control one or more of these cellular mechanisms could lead to altered cell proliferation and growth, which in some cases can trigger a compensatory mechanism. For example, in leaves of flowering plants, the total abundance of cells that results from abnormal mitotic cell division would induce corresponding changes in post-mitotic cell expansion, to ensure final leaf size is not significantly changed compared with that expected from variation in the quantity of leaf cells (Horiguchi and Tsukaya, 2011).

Recent studies have enhanced our understanding of the molecular mechanisms underlying organ size determination (Gonzalez *et al.*, 2012; Hepworth and Lenhard, 2014; Czesnick and Lenhard, 2015). Notably, the ubiquitin-proteasome pathways have been revealed to play a significant role in the regulation of cell-cycle progression during plant organ morphogenesis (Vierstra, 2003). The F-box protein AtFBX92 represses leaf growth by influencing the cell division rate during the early stages of leaf development in Arabidopsis (Baute *et al.*, 2017). Another F-box protein, FBOX-LIKE17 (FBL17), accelerates cell proliferation and endoreduplication by forming an Skp1/Cullin/F-box (SCF) E3 ubiquitin ligase complex that targets KIP-RELATED PROTEIN2 (KRP2) for degradation (Noir *et al.*, 2015). The peptidase DA1 is activated by the RING E3 ligases DA2 and ENHANCER OF DA1/BIG BROTHER (EOD1/BB )via mono-ubiquitination, thereby negatively regulating final organ size by destabilizing various positive growth factors (Disch *et al.*, 2006; Li *et al.*, 2008; Xia *et al.*, 2013; Du *et al.*, 2018; Dong *et al.*, 2017; Vanhaeren *et al.*, 2020). AtRPT2a, being a subunit of the 26S proteasome, represses organ size by reducing both cell endoreduplication and expansion (Sonoda *et al.*, 2009). It was also shown that STERILE APETALA-PEAPOD-KIX-TOPLESS (SAP-PPD-KIX-TPL), KIX-PEAPOD-MYELOCYTOMATOSIS-GRF-INTERACTING FACTOR 1 (KIX-PPD-MYC-GIF1) and STERILE APETALA-PEAPOD-NOVEL INTERACTOR OF JAZ-TOPLESS (SAP-PPD-NINJA-TPL) modules govern lateral organ size, seed size and leaf shape in Arabidopsis (White, 2006; Gonzalez *et al.*, 2015; Wang *et al.*, 2016; Baekelandt *et al.*, 2018; Li *et al.*, 2018; Liu *et al.*, 2020). The F-box protein SAP integrates with other subunits to form the SCF^SAP^ E3 ubiquitin ligase; this marks the PPD2-KIX8/9 repressor complex for degradation to determine the final organ size, or for degradation of the PPD2-NINJA repressor complex to ensure that a normal leaf blade curvature develops in Arabidopsis (Byzova *et al.*, 1999; Wang *et al.*, 2016; Baekelandt *et al.*, 2018; Li *et al.*, 2018). Either knock down or knock out of *PPD2* and its orthologs, or the loss-of-function of *KIX8*/*9* and its orthologs, gave rise to an enlarged and dome-shaped leaf, but mutation of *SAP* and its orthologs led to a reduction in the size of lateral organs (White 2006; Gonzalez *et al.*, 2015; Ge *et al.*, 2016; Wang *et al.*, 2016; Naito *et al.*, 2017; Kanazashi *et al.*, 2018; Li *et al.*, 2018; Yang *et al.*, 2018).

Various leaf movements have been observed in higher plants in response to endogenous and environmental signals. These intriguing behaviors are proposed to provide advantageous strategies for plant adaptation, survival, and evolution ( Darwin, 1880; Satter *et al.*, 1990; Koller, 2011; Mancuso and Shabala, 2015; Minorsky, 2019). Reversible leaf movement relies on the pulvinus, a specialized motor organ in higher plants (Satter *et al.*, 1990; Coté, 1995; Ueda and Nakamura, 2007). Yet, surprisingly, the molecular mechanisms underlying pulvinus development are still poorly understood. It is known that ELONGATED PETIOLULE1 (ELP1), a plant-specific lateral organ boundaries domain (LBD) transcription factor, determines the pulvinus identity in legumes. Disruption of *ELP1* expression or that of its orthologs in related legume species completely abolished both pulvinus development and leaf movement (Marx, 1987; Kawaguchi, 2003; Chen *et al.*, 2012; Zhou *et al.*, 2012). The gene *Glycine max Increased Leaf Petiole Angle1* (*GmILPA1*) encodes an APC8-like protein that is a component of the APC/C complex and able to interact with GmAPC13a to promote motor cell division and growth in soybean (*Glycine max*). The mutation of *GmILPA1* results in defective pulvinus formation and diminishes leaf movement (Gao *et al.*, 2017).

Here we report on the identification and characterization of the *Medicago truncatula* mutant *mini organ1* (*mio1*), which has a decreased organ size and a defective pulvinus. *MIO1* encodes an F-box protein, SLB1, which has been shown to control lateral branching in *M. truncatula* (Yin *et al.*, 2020). *MIO1*/*SLB1* is the ortholog of *SAP* and highly expressed in both leaf and floral primordia. The transformation of *MIO1*/*SLB1* into the *mio1* mutant completely rescued the latter’s traits of smaller organ size and defective pulvinus. Protein-protein interaction assays revealed that MIO1/SLB1 could directly interact with *Medicago truncatula Arabidopsis* SKP (MtASK), and with BIG SEEDS1 (BS1) via its C terminal WD40 repeat domain. The protein degradation assay indicated that BS1 could be degraded by MIO1/SLB1 in an MG132-sensitive manner. Loss-of-function of *MIO1*/*SLB1* induced down-regulation of expression of the cell-cycle marker genes. Our work reveals that MIO1/SLB1 plays a key role not only in the determination of lateral organ size, which would be valuable for improving biomass production and yield of legume crops and forages, but also in the regulation of normal pulvinus development that is necessary for leaf movement in *M. truncatula*.

## Materials and methods

### Plant materials and growth conditions

Arabidopsis (Col-0) and *M. truncatula* (R108) plants were grown in a chamber and greenhouse, respectively, under the following conditions: a 16 h/8 h day/night photoperiod, 150 μE m^-2^ s^-1^ light intensity, temperatures of 22 °C/18 °C at day/night, and under 70% humidity. The *mio1-1* mutant was backcrossed with the wild type (WT) for three generations, and BC3F2 (the F_2_ population which was backcrossed with WT three times) were used for phenotypic and genetic analyses.

### Phenotypic analysis

The length, width, and area of leaflets were measured using ImageJ. For the time-course analysis of leaf development, a juvenile leaf was imaged daily over a 12-day period with a digital camera (Nikon); data were collected with ImageJ. To analyse the leaf epidermal cell area, stomatal index, and for the follow-up investigation of cell area, images of epidermal cells were captured under a microscope (BX63, Olympus, Japan). From these, the cell area and stomatal index were determined using ImageJ.

### Whole-genome resequencing and molecular cloning of *MIO1*/*SLB1*

The heterozygous plants of *mio1-1*, *mio1-2*, and *mio1-3* were backcrossed with WT (R108) for two generations. The ensuing homozygous mutant plants of *mio1-1*, *mio1-2*, and *mio1-3* were then used for whole-genome resequencing (next-generation sequencing). Specifically, their sequence data was analysed with the ITIS (Identification of Transposon Insertion Sites) tool, to identify all *Tnt1* insertion sites as described previously (Jiang *et al.*, 2015). Both PCR and RT–PCR were used for generating cosegregation analysis and confirmation of *Tnt1* insertions responsible for the mutant phenotype. The primers used are listed in Supplementary Table S1.

### Phylogenetic analysis and sequence alignment

The protein homologs of MIO1/SLB1 were identified from phytozome database (https://phytozome.jgi.doe.gov/pz/portal.html#). All the candidates were verified to contain the conserved F-box and WD40 repeat motif. Next, phylogenetic trees were constructed by using the neighbor-joining algorithm implemented in MEGA5, with 1000 bootstrap replications performed. The sequence alignment of MIO1/SLB1 and its homologs was carried out with DNAMAN software.

### Scanning electron microscope (SEM) analysis

The pulvini from the *mio1-1*, *proMIO1::MIO1*/*mio1-1* transgenic plants, and WT were collected. The collected tissues were subjected to vacuum infiltration in FAA fixative solution for 30 min and then kept at 27 °C overnight. The fixed tissues were dehydrated in a graded ethanol series (45%, 55%, 65%, 75%, 85%, 90%, 95%, each concentration for about 1 h), and ending with 100% ethanol overnight. The dehydrated tissues were dried until critical-point in liquid CO_2_ with a critical point drier (Samdri-PVT-3D, Tousimis, USA), mounted on aluminum stubs, dissected with the stereomicroscope (SZX16, Olympus, Japan), sputter-coated with gold, and examined under SEM (SIGMA 300, Zeiss, Germany).

### Paraffin sectioning

The fixed and dehydrated pulvini samples were embedded in paraplast (EG1150 H, Leica, Germany. These plant tissues were sectioned at an embedder (RM2235, Leica, Germany), to an 8 μm thickness and stained with toluidine blue. Their images were captured with a microscope (Olympus BX63M).

### RNA *in-situ* hybridization

Shoot apices of three-week-old and 10-week-old WT plants were paraffin embedded and sectioned. These sections were hybridized with a digoxigenin-labeled anti-sense probe, using the sense probe to serve as the control. The hybridization signal was visualized under a microscope (Olympus BX63M).

### Plasmid construction and transformation

The 4.7 kb promoter sequence of *MIO1*/*SLB1* was generated from genome sequence and cloned into the pCAMBIA3301 vector to obtain the *proMIO1::GUS* construct. The coding sequence of *MIO1*/*SLB1* was inserted into the *proMIO1::GUS* vector in which the *GUS* gene was replaced with *MIO1*/*SLB1* coding sequence, to obtain the *proMIO1::MIO1* construct. The coding sequence of *MIO1*/*SLB1* was cloned into the pCAMBIA3301 vector to generate the *35S::MIO1* construct. For the construction of *35S::GFP-MIO1*, the coding sequence of *MIO1*/*SLB1* was cloned into pCAMBIA3301 To generate *35S::MIO1-GFP*, the *MIO1*/*SLB1* coding sequence was inserted into pYS22. To derive the plasmids used for the yeast two-hybrid (Y2H) assay, the full-length *MIO1/SLB1* coding sequence and the truncated sequence that corresponds to the F-box or WD40 repeat domain of MIO1/SLB1 were inserted into the bait vector pGBKT7 (Clontech, USA). Similarly, the coding sequences of *BS1, MtASK,* and *MtKIX* were inserted into the prey vector pGADT7. For the bimolecular fluorescence complementation (BiFC) assay, the *MIO1/SLB1* coding sequence was inserted into the pFGC-YN173 vector while *MtASK* and *BS1* were inserted into the pFGC-YC155 vector. To obtain a plasmid that expresses the GST-MIO1 WD40 fusion, the truncated coding sequence of Medtr5g097060 corresponding to the WD40 repeat domain was cloned into pGEX4T-1 that harbored the *GST* coding sequence. Similarly, the coding sequence of *BS1* was inserted into pET28a that contained the His tag, to generate the His-BS1 fusion protein. All the primers used for the above are listed in the Supplementary Table S1.

### Sub-cellular localization

The *35S::GFP-MIO1* and *35S::MIO1-GFP* constructs were transformed into the *Agrobacterium tumefaciens* EHA105 strain, and then infiltrated into tobacco (*Nicotiana benthamiana*) leaves. The MIO1-GFP and GFP-MIO1 signals were examined under a confocal laser scanning microscope (Olympus, FV1000).

### RNA isolation, RT–PCR, and quantitative RT–PCR

Total RNA was isolated from plant tissues with a plant total RNA isolation kit (Generay, China). The quantity and quality of extracted RNA were assessed with Nanodrop 2000 (Thermo Scientific, USA). Next, the cDNA synthesis was performed with PrimeScript^TM^ RT reagent kit (Takara, Japan), following the manufacturer’s instructions. For RT–PCR, the cDNA was amplified in 30 cycles and the products were separated on a 1.5% sepharose gel. The *MtActin* gene was used as an internal control. Quantitative RT–PCR analysis was conducted by LightCycler 480 engine (Roche, Switzerland), for which both *MtActin* and *MtGAPDH* served as internal controls. The primers for this can be found in Supplementary Table S1.

### GUS staining assay

Leaf samples of the *proMIO1::GUS* transgenic plants were collected and subjected to vacuum infiltration in a GUS staining solution, and then incubated overnight at 37 °C overnight. The WT served as the negative control.

### Yeast two-hybrid (Y2H) assay

The constructed bait and prey plasmids were co-transformed into the Y2H Gold Yeast Strain (Clontech) and cultured on SD/-Leu-Trp media, for three days, at 30 °C. The ensuing interactions were detected on selective media -4 (SD/-Ade/-His/-Leu/-Trp).

### Bimolecular fluorescence complementation (BiFC) assay

The *A. tumefaciens* strains harboring the N-terminal YFP fused and C-terminal YFP fused constructs were mixed in a 1:1 ratio and co-infiltrated into tobacco leaves. Fluorescence signals were examined at 48 h post-infiltration under a confocal laser scanning microscope (Olympus, FV1000).

### Pull-down assay

The bacterial lysates containing the GST-MIO1WD40 fusion protein and those lysates containing the His-BS1 fusion proteins were mixed and incubated with glutathione sepharose beads (GE Healthcare), for 2 h, at 4 °C. The protein was separated using 10% SDS-PAGE. The ensuing interaction was detected by immunoblotting with an anti-His antibody. GST was used as a negative control.

### Protein degradation assay

The His-BS1 fusion protein was expressed in *E. coli*, then extracted and purified with a His-tag protein purification kit (Beyotime, China) according to the manufacturer’s instructions. Total protein from the WT, *mio1* mutant, and *MIO1* overexpression lines (WT *35S::MIO1*) was extracted from an equal amount of leaf tissue. Then the purified His-BS1 protein was mixed with the total protein solution, in a 3:1 volume ratio. For the control, the total protein was first pre-treated with MG132 before mixing it with His-BS1. All the mixtures were incubated at 4 °C with gentle shaking applied. Samples were removed at different time points (0 min and 30 min), the catalytic reaction was stopped by boiling for 5 min at 95 °C with SDS loading buffer. The denatured protein was separated on 10% SDS-PAGE and detected by an immunoblot analysis using anti-His antibodies (Transgene, China). Equal amounts of the total proteins were used as loading controls.

## Results

### Isolation and characterization of the *M. truncatula mio1* mutant

**Fig. 1.**
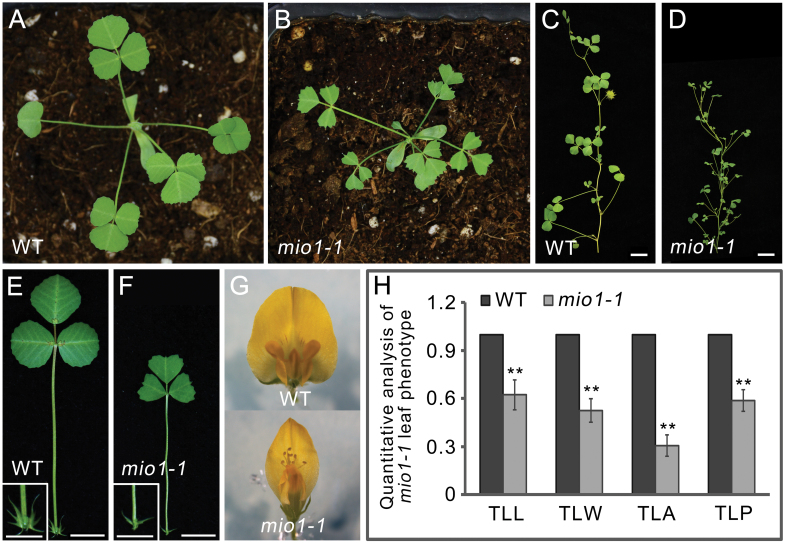
Phenotype comparisons between the wild type (WT) and *mio1* mutant. (A, B) Four-week-old seedlings of the WT (A) and *mio1-1* mutant (B). Scale bar=2 cm. (C, D) Branch of a 12-week-old plant of the WT (C) and *mio1-1* mutant (D). Scale bar=2 cm. (E, F) The fifth compound leaf of five-week-old seedlings of the WT (E) and *mio1-1* mutant (F). Insets provide a close-up view of the stipules. Scale bar=1 cm for leaves, 0.5 cm for stipules. (G) Flower (open petal) of the WT (upper) and *mio1-1* mutant (bottom) plants. Scale bar=2 mm. (H) Comparison of the terminal leaflet length (TLL), terminal leaflet width (TLW), terminal leaflet area (TLA), and terminal leaflet perimeter (TLP) of the WT and *mio1-1* mutant plants. Values indicate the mean±SD (*n*=3 biological replicates, with 20 plants per replicate); asterisks indicate significant differences from the WT (**P<0.01; Student’s *t*-test).

To identify the key regulators that control the organ size of legumes, we screened the *Tnt1* retrotransposon insertion mutant population of the model legume *M. truncatula* (R108) for any organ size mutants. Three independent mutant lines—*mini organ1* (*mio1*)*-1*, *mio1-2*, and *mio1-3*—exhibiting similarly decreased organ size, were isolated. A genetic analysis confirmed that they were allelic mutants. Then the *mio1-1* mutant was backcrossed with the WT plants. In the BC1F2 population, the plants displaying the WT-like and mutant phenotype showed a 3:1 (149:51) segregation ratio; this indicated that the mutant phenotype was caused by the mutation of a single recessive gene. The *mio1-1* was backcrossed with WT for three generations, and then the BC3F2 plants were used for phenotypic analysis. Compared with the WT, the *mio1* mutant was evidently reduced in its plant and organ size in both its vegetative (Fig. 1A, B) and reproductive phases (Fig.1C, D). The sizes of the representative lateral organs, namely the leaves and flowers, were markedly smaller in the *mio1* mutant than the WT (Fig. 1E-G). The terminal leaflet length (TLL), terminal leaflet width (TLW), terminal leaflet area (TLA), and terminal leaflet perimeter (TLP) of the *mio1* mutant exhibited significant (*P*<0.01) reductions compared with those of the WT plants (Fig. 1H). These results indicated that a functional *MIO1* gene is essential for proper organ size regulation in *M*. *truncatula*.

### Molecular cloning and characterization of *MIO1*/*SLB1*

**Fig. 2.**
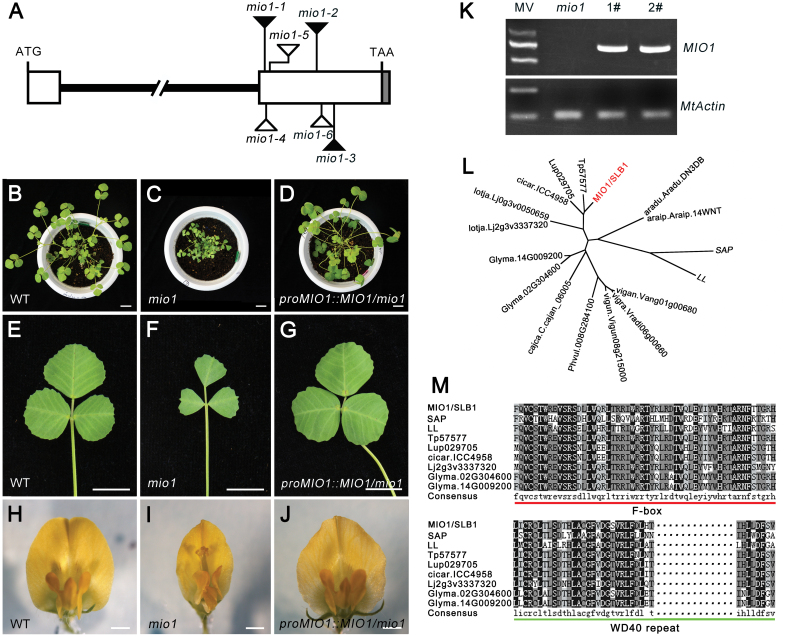
Molecular cloning and characterization of *MIO1*/*SLB1*. (A) The *MIO1*/*SLB1* gene structure and *Tnt1* retrotransposon insertion sites in the *mio1* mutant alleles. White and grey boxes represent the exon and 3**’** UTR, respectively, and the bold line represents the intron. Triangles indicate the *Tnt1* insertion sites of *mio1* mutant alleles, while the black and white ones indicate mutants obtained from forward and reverse screening, respectively. (B-J) Genetic complementation of the *mio1* mutant. Five-week-old seedlings of the WT (B), *mio1* mutant (C), and complemented *mio1* line *proMIO1::MIO1*/*mio1* (D). The fifth trifoliate leaf of five-week-old seedling of WT (E), *mio1* mutant (F), and complemented *mio1* line *proMIO1::MIO1*/*mio1* (G). Flower (open petal) of the WT (H), *mio1* mutant (I), and complemented *mio1* line *proMIO1::MIO1*/*mio1* (J). Scale bars=2 cm (B-D), 1 cm (E-G), 2 mm (H-J). (K) RT–PCR analysis of *MIO1*/*SLB1* gene expression in the complemented *mio1* lines. The expression of *MIO1*/*SLB1* was restored in the independent complemented line 1 (1#) and line 2 (2#). Total RNA was extracted from the shoot apices of five-week-old seedlings. MW, molecular weight markers; *MtActin* was used as an internal control. (L) Phylogenetic analysis of MIO1/SLB1 and its closely related homologs. SAP from *Arabidopsis thaliana,* LL from *Cucumis sativus*, with other homologs from several Fabaceae species including *Trifolium pratense*, *Lupinus angustifolius*, *Cicer arietinum*, *Lotus japonicus*, *Glycine max*, *Cajanus cajan*, *Phaseolus vulgaris*, *Vigna unguiculata, Vigna radiata*, *Vigna angularis*, *Arachis ipaensis*, and *Arachis duranensis*. (M) Amino acid sequence alignment of MIO1/SLB1 and its closely related homologs. The red and green underlining indicates the F-box and WD40 repeat domain, respectively. SAP from *A. thaliana*, LL from *C. sativus*, Tp57577 from *T. pratense*, Lup029705 from *L. angustifolius*, cicar.ICC4958 from *C. arietinum*, Lj2g3v3337320 from *L. japonicus*, and Glyma.02G304600 and Glyma.14G009200 from *G. max*. Dotted lines indicate the abridged conserved amino acids.

The *MIO1* gene was cloned with the ITIS tool by identifying the *Tnt1* retrotransposon insertion that caused the mutant phenotype (Jiang *et al.*, 2015). The *mio1-1*, *mio1-2*, and *mio1-3* mutants were backcrossed with WT, after which the mutant plants of BC2F2 were used for whole genome resequencing (He *et al.*, 2020). The *Tnt1* insertion sites were then isolated, by analysing the resequencing data with the ITIS tool, as previously described (Jiang *et al.*, 2015). These isolation results showed that all three mutant alleles were homozygous for the *Tnt1* insertions at the same ORF (open reading frame) of the Medtr5g097060 locus at different sites (Fig. 2A; Supplementary Table S2). These insertions co-segregated with the mutant phenotype, according to the PCR-based genotyping results of homozygous recessive mutants of the BC2F2 population, in all the three alleles. In contrast with WT, the transcript of Medtr5g097060 could not be detected in the *mio1* mutant by RT–PCR analysis (Supplementary Fig. S1). Collectively, these data suggested that the Medtr5g097060 locus defines the gene corresponding to *MIO1*.

To further confirm that the Medtr5g097060 locus corresponds to the *MIO1* gene, we reverse-screened the *Tnt1* insertion population for additional alleles, and also carried out genetic complementation. Three new mutant lines harboring the *Tnt1* insertions at different sites of the Medtr5g097060 locus were isolated; they displayed similar phenotypes to the *mio1-1* mutant and were named *mio1-4*, *mio1-5*, and *mio1-6* (Fig. 2A; Supplementary Table S2). Subsequently, the coding sequence (CDS) of *MIO1* driven by its native promoter was introduced into the *mio1-1* mutant via *A. tumefaciens*-mediated transformation. The transgenic plants resulted in full complementation of the mutant phenotype in two independent complemented lines (Fig. 2B-D). Importantly, in these lines, the reduced leaf size trait was completely rescued (Fig. 2E-G), and the decreased floral organ size was also recovered (Fig. 2H-J), to match those of the WT phenotype. The expression of *MIO1* was evidently restored in these complemented plants (Fig. 2K). The Medtr5g097060 locus has been reported to also encode the *SMALL LEAF AND BUSHY1* (*SLB1;*Yin *et al.*, 2020); we confirmed that the *MIO1* gene was allelic to *SLB1* by comparing the gene sequence and mutant phenotypes. Taken together, these results confirmed that the Medtr5g097060 locus corresponds to *MIO1* that is allelic to the *SLB1* gene and required for organ size determination in *M*. *truncatula*.

Phylogenetic analysis revealed MIO1/SLB1 as the ortholog of STERILE APETALA (SAP) and LITTLE LEAF (LL; Fig. 2L; Supplementary Fig. S2), the key organ size regulators in Arabidopsis and cucumber (*Cucumis sativus*), respectively (Wang *et al.*, 2016; Yang *et al.*, 2018). Multiple amino acid sequence alignments of MIO1/SLB1 with its orthologs indicated that they shared the highly conserved N-terminal F-box and C-terminal WD40 repeat domains (Fig. 2M; Supplementary Fig. S3). All of these features suggested that *MIO1*/*SLB1* encodes an F-box protein. F-box proteins are known to form the SCF E3 ubiquitin ligase with ASK and Cullin through its N-terminal F-box domain, and recognize specific substrates for ubiquitination through its C-terminal domain, just like the WD40 repeat domain (Vierstra, 2003; Wang *et al.*, 2016).

### Ectopic expression of *MIO1*/*SLB1* rescues the mutant phenotype of Arabidopsis *sod3-3*

**Fig. 3:**
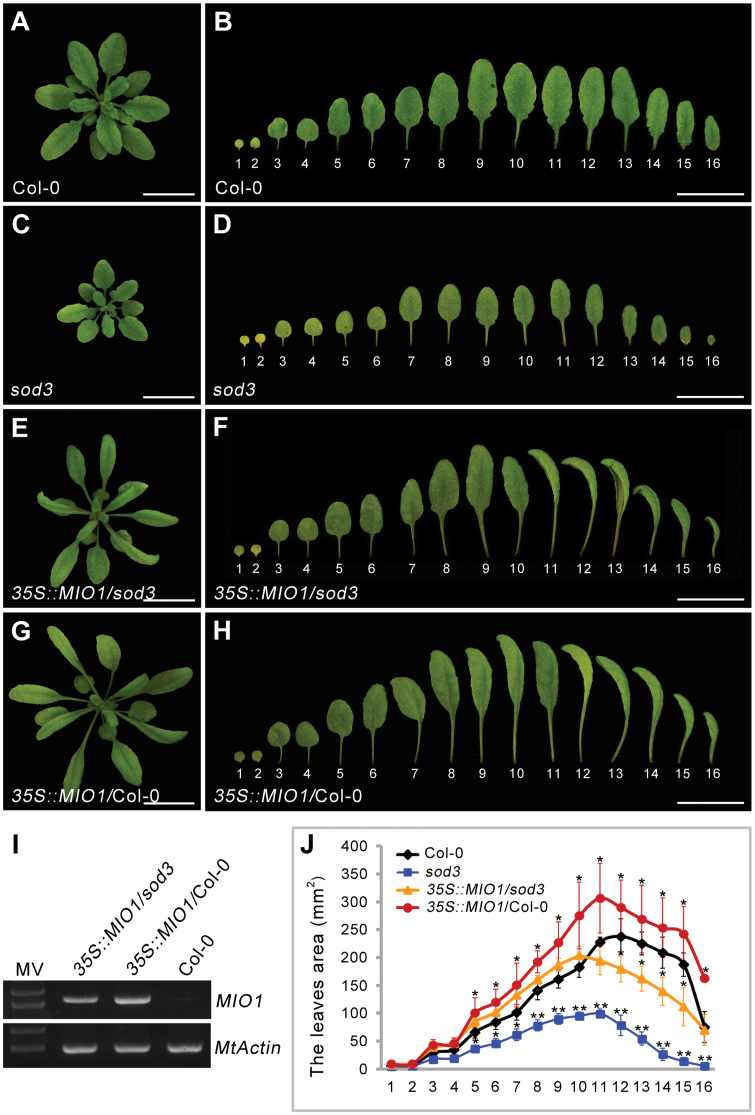
Ectopic expression of *MIO1*/*SLB1* in Arabidopsis *sod3-3* (*35S::MIO1*/*sod3*) mutant and wild type (*35S::MIO1*/ Col-0) plants. (A-H) Five-week-old seedlings of Col-0 (A), *sod3****-****3* mutant (C), *35S::MIO1*/*sod3* (E), and *35S::MIO1*/Col-0 (G). Scale bar=2cm. The first to sixteenth leaves of five-week-old seedlings of Col-0 (B), *sod3****-****3* mutant (D), *35S::MIO1*/*sod3* (F), and *35S::MIO1*/Col-0 (H). Scale bar=2cm. (I) RT–PCR analysis of *MIO1*/*SLB1* expression in the complemented *sod3* line and Col-0 plants. (J) The first to sixteenth leaves area of five-week-old seedlings of the Col-0, *sod3****-****3* mutant, *35S::MIO1*/*sod3*, and *35S::MIO1*/Col-0. The leaves 1 and 2 are cotyledons. Values indicate the mean±SD (*n*=3 biological replicates; 10 plants per replicate). Asterisks indicate significant differences from the WT (**P<0.01, *P<0.05; Student’s *t*-test).

The Arabidopsis *MIO1*/*SLB1* ortholog, *STERILE APETALA* (*SAP*)/*SUPPRESSOR OF DA1* (*SOD3*), is known to positively regulate organ size during plant growth and development (Wang *et al.*, 2016). Loss-of-function of *SAP* gave rise to decreased organ size in Arabidopsis (Wang *et al.*, 2016). To investigate whether *MIO1*/*SLB1* performs a similar function as *SAP* for organ size control, the CDS of *MIO1*/*SLB1* driven by the cauliflower mosaic virus 35S (*CaMV35S*) promoter was introduced into the Arabidopsis *sod3****-****3* mutant (*35S::MIO1*/*sod3****-****3*) and the corresponding WT (*35S::MIO1*/Col-0). The smaller organ size of *sod3****-****3* was rescued in *35S::MIO1*/*sod3****-****3* transgenic plants (Fig. 3A-F, J). Furthermore, the *35S::MIO1*/Col-0 transgenic lines exhibited increased organ size compared with Col-0 (Fig. 3G, H, J). Interestingly, most of the transgenic plants had twisted and dome-shaped leaves (Fig. 3F, H). This phenotype was similar to that reported for the Arabidopsis *ppd* mutant (White, 2006). The expression of *MIO1*/*SLB1* was detected in both *35S::MIO1*/*sod3****-****3* and *35S::MIO1*/Col-0 transgenic plants (Fig. 3I). These results suggested that *MIO1*/*SLB1* and *SAP* play a conserved role in organ size regulation.

### Expression pattern of *MIO1*/*SLB1* and sub-cellular localization of MIO1/SLB1

**Fig. 4.**
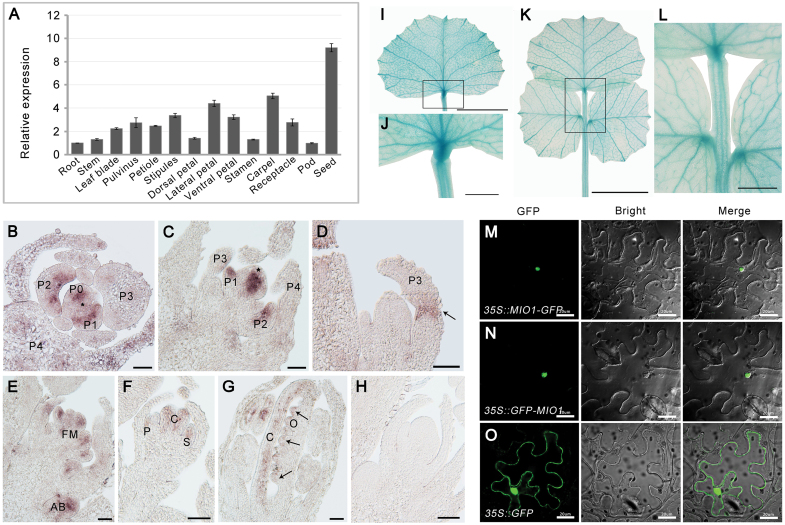
Expression pattern of *MIO1*/*SLB1* and sub-cellular localization of MIO1/SLB1. (A) Expression of *MIO1*/*SLB1* in different plant tissues analysed by qRT–PCR, with *MtActin* used as an internal control. Values indicate the mean±SD (*n*=3). (B-H) RNA *in situ* hybridization of *MIO1*. Cross (B) and longitudinal (C and D) sections of shoot apices from three-week-old wild type (WT) seedlings (vegetative stage) that were hybridized with the *MIO1*/*SLB1* anti-sense probe. Longitudinal sections of shoot apices from 10-week-old WT plants (E), floral meristem (F), and 1 mm flower bud (G) that were hybridized with the *MIO1*/*SLB1* anti-sense probe. P+number, plastochron; FM, floral meristem; AB, axillary buds; P, petal; C, carpel; S, stamen; O, ovule; asterisks denote the shoot apical meristem. The black arrow points to the pulvinus primordium. The *MIO1*/*SLB1* sense probe was used as the control (H). Scale bar=50 μm. (I-L) GUS staining of single leaf (I and J) and trifoliate leaf (K and L) of four-week-old seedlings of *proMIO1::GUS* transgenic plants. J and L are the close-up views of the framed area in I and K, respectively. Scale bars=1 cm (I and K), 2 mm (J and L). (M-O) Sub-cellular localization of the MIO1-GFP (M) and GFP-MIO1 (N) fusion proteins. Free GFP driven by the CaMV35S promoter was used as the control (O). Scale bar=20 μm.

Quantitative RT-PCR revealed that *MIO1*/*SLB1* was highly expressed in leaves, floral organs, and immature seeds (Fig. 4A; Supplementary Fig. S4). Further analysis with RNA *in-situ* hybridization showed that the *MIO1*/*SLB1* transcripts mainly accumulated in the early leaf primordia (P0-P2; Fig. 4B, C), pulvinus primordia (Fig. 4D), floral meristem, axillary bud (Fig. 4E), and petal meristem, developing carpel, and ovule (Fig. 4F, G), with no signals detected in the control which hybridized with a sense probe (Fig. 4H). To further confirm this, but using a different approach, we introduced the *GUS* reporter gene driven by the *MIO1*/*SLB1* promoter into WT (*proMIO1::GUS*). GUS signals were detected in both single and trifoliate leaves, and the signals were stronger in vascular tissue and pulvinus (Fig. 4I-L). Taken together, the above expression pattern suggested a crucial role for *MIO1*/*SLB1* during lateral organ morphogenesis in *M. truncatula*.

To determine its sub-cellular localization, we transiently expressed *MIO1/SLB1* fused with a green fluorescent protein (*MIO1*-GFP or *GFP*-MIO1) under the control of a *35S* promoter in tobacco leaves. Both the MIO1-GFP and GFP-MIO1 fusion proteins were localized to the nuclei of tobacco leaf epidermal cells (Fig. 4M, N). Free GFP driven by the *35S* promoter was used as the negative control (Fig. 4O).

### MIO1/SLB1 positively regulates primary cell division

**Fig. 5.**
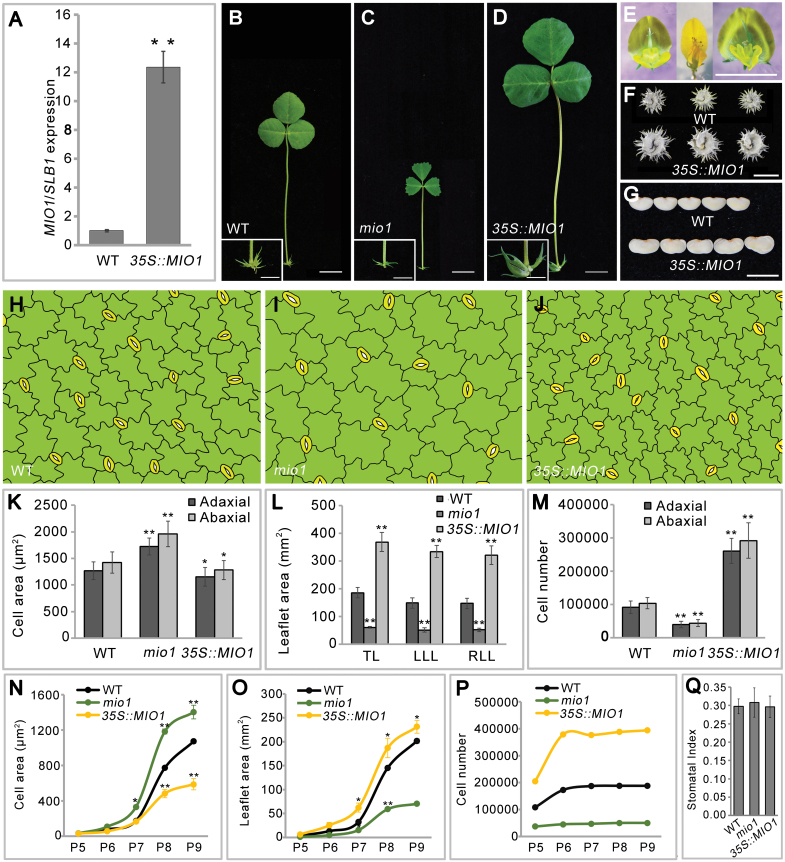
*MIO1*/*SLB1* positively regulates primary cell division during leaf development. (A) *MIO1*/*SLB1* transcript abundance in the wild type (WT) and *MIO1-*overexpressing line, with *MtActin* used as an internal control. Values indicate the mean±SD (*n*=3); asterisks indicate significant differences with respect to the WT (**P<0.01; Student’s *t*-test). (B-D) The fifth compound leaf of five-week-old seedlings of WT (B), *mio1* mutant (C), and *MIO1*-overexpressing line *35S::MIO1* (D). Insets are close-up views of stipules. Scale bars=1 cm for leaves, 0.5 cm for stipules. (E) Flower (open petal) of the WT (left), *mio1* mutant (middle), and *MIO1*-overexpressing (right) plants. Scale bar=1 cm. (F, G) The pod (F) and seed (G) of the WT (upper) and *MIO1*-overexpressing (bottom) plants. Scale bars=1 cm (E), 0.5 cm (F). (H-J) The epidermal cell outlines for the abaxial epidermis of a mature leaflet of the WT (H), *mio1* mutant (I), and *MIO1-*overexpressing line (J). These pictures were outlined with Photoshop software based on photos from the microscope observations. Green and yellow-colored cells represent epidermal and guard cells, respectively. (K) The leaf epidermal cell area of the WT, *mio1* mutant, and *MIO1*-overexpressing line. Values indicate the means±SD (*n*=3 biological replicates, with 10 plants per replicate); asterisks indicate significant differences from the WT (*P<0.05, **P<0.01; Student’s *t*-test). (L) Leaflet area of the terminal leaflet (TT), left lateral leaflet (LLL), and right lateral leaflet (RLL) of the WT, *mio1* mutant, and *MIO1*-overexpressing line. Values indicate the mean±SD (*n*=3 biological replicates, with 10 plants per replicate); asterisks indicate significant differences from the WT (**P<0.01; Student’s *t*-test). (M) Average total number of epidermal cells for the adaxial epidermis of a mature leaflet of the WT, *mio1* mutant, and *MIO1*/*SLB1* overexpressing-line. Values indicate the mean±SD (*n*=3 biological replicates, with 10 plants per replicate); asterisks indicate significant differences from the WT (**P<0.01; Student’s *t*-test). (N-P) Statistical analysis of terminal leaflet abaxial epidermal cell area (N), terminal leaflet area (O), and total number of epidermal cells for the abaxial epidermis (P) of P5-P9 (plastochron 5 to plastochron 9) of WT, *mio1* mutant, and *MIO1*-overexpressing line. Values indicate the mean±SD (*n*=3 biological replicates, with 10 plants per replicate); asterisks indicate significant differences from the WT (*P<0.05; **P<0.01, Student’s *t*-test). (Q) The stomatal index for the abaxial leaflet epidermis of the WT, *mio1* mutant, and *MIO1*-overexpressing line. Values indicate the mean±SD (*n*=3 biological replicates, with 20 plants per replicate); asterisks indicate significant differences from the WT (*P<0.05, **P<0.01; Student’s *t*-test).

Loss-of-function mutation of *MIO1*/*SLB1* resulted in reduced lateral organ size compared with the WT (Fig. 5B, C; Supplementary Fig. S5A, B).

To gain insight into the mechanism by which *MIO1*/*SLB1* functions to regulate organ size, *MIO1*-overexpressing plants were generated by introducing the *35S::MIO1* construct into WT. Ten independent transgenic lines exhibited enlarged organs, especially for leaf (Fig. 5D; Supplementary Fig. S5C), floral organ (Fig. 5E), pod (Fig. 5F), and seed (Fig. 5G); these phenotypes were very similar to the reported *M. truncatula bs1* mutant (Ge *et al.*, 2016). *MIO1*/*SLB1* expression increased by more than twelve-fold when compared with the WT in the *MIO1*-overexpressing transgenic lines (Fig. 5A; Supplementary Fig. S6).

Final organ size in plants is determined by a complex coordination of cell division and expansion (Gonzalez *et al.*, 2012). Microscopic examination of mature leaf epidermal cells revealed that cell size was opposite to MIO1/SLB1 activity: epidermal cell size increased in the *mio1* mutant while it decreased in the *MIO1*-overexpressing plants in comparison with the WT (Fig. 5H-J; Supplementary Fig. S5D-F). Using the measurements of epidermal cell area (Fig. 5K) and leaflet area (Fig. 5L), we calculated the total number of epidermal cells per given leaflet (Fig. 5M). On average, this value for *MIO1*-overexpressing plants increased by more than two-fold compared with WT on both adaxial and abaxial sides (Fig. 5M). However, the number of cells in the *mio1* mutant was only about half that of the WT (Fig. 5M). These results suggested that *MIO1*/*SLB1* augments leaf size mainly by increasing the abundance of cells (and not their individual area).

The follow-up investigation of abaxial epidermal cell area (Fig. 5N), leaflet area (Fig. 5O), and the calculated total number of cells per leaflet (Fig. 5P) indicated that differences in leaf epidermal cell size among the *MIO1*-overexpressing, *mio1* mutant, and WT plants had its origin around the P6 leaf development stage, and this became magnified in later stages. Differences in cell abundance began to emerge at P5, and reach a plateau at P6 (Fig. 5N, P). All of these findings implicated MIO1/SLB1 in influencing primary cell division during leaf growth and development. Although the cell size and number differed, the leaf epidermal cell pattern (Fig. 5H-J; Supplementary Figs S5D-F;  S7) and stomatal index (Fig. 5Q) were similar among the *MIO1*-overexpressing, *mio1* mutant, and WT plants, further pointing to the promotion of primary cell proliferation by MIO1/SLB1 activity. The time point at which leaflet length, width, and area peaked in size was likely delayed for *MIO1*-overexpressing plants and occurred sooner for the *mio1* mutant, when compared with the WT, but the duration of leaflet expansion showed no clear differences (Supplementary Fig. S8). This indicated that meristemoid cell division is not altered when *MIO1*/*SLB1* is overexpressed or knocked out. Taken together, these results suggested that MIO1/SLB1 positively regulates organ size mainly by promoting the process of primary cell proliferation during plant development.

### F-box protein MIO1/SLB1 regulates the stability of BS1 *in vitro*

Previous reports demonstrated that SAP interacts with ASK1/2 and CUL1 (Cullin1), to form a conserved SCF E3 ubiquitin ligase, which targets the repressor PPD for degradation to thereby enlarge the size of organs (Wang *et al.*, 2016). The loss-of-function of BS1, the ortholog of PPD in *M. truncatula*, also led to enlarged lateral organs similar to the *ppd* mutant (Ge *et al.*, 2016). These clues suggest that the role of MIO1/SLB1 in regulating organ size may involve the conserved ubiquitin-proteasome at its core.

**Fig. 6.**
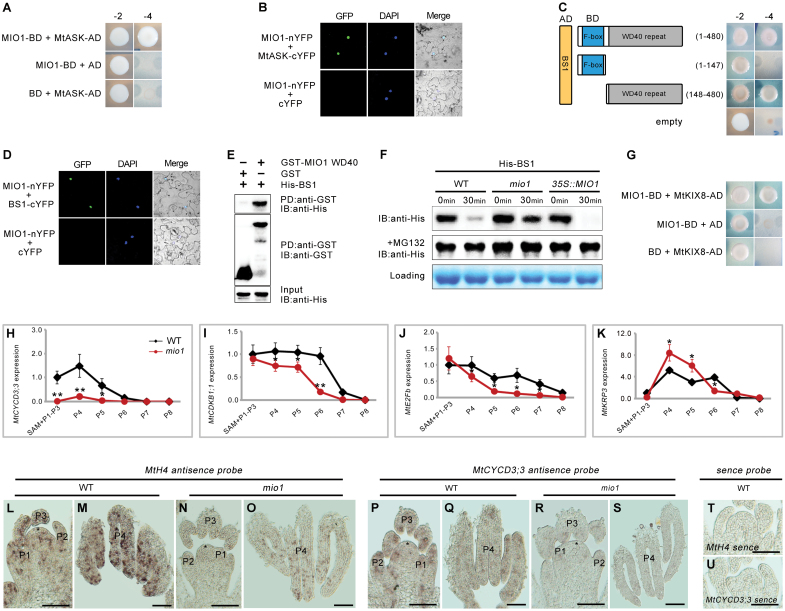
F-box protein MIO1/SLB1 physically interacts with, and regulates BS1 stability. (A) Yeast two-hybrid (Y2H) assay showing the interaction between MIO1/SLB1 and MtASK. -2, SD/-Leu/-Trp; -4, SD/-Ade/-His/-Leu/-Trp. (B) Bimolecular fluorescence complementation (BiFC) assay showing the interaction between MIO1/SLB1 and MtASK in the nuclei of tobacco (*Nicotiana benthamiana*) leaf epidermal cells. MIO1-nYFP and MtASK-cYFP were coexpressed in the leaves of tobacco; DAPI signals indicate the nuclei. (C) Y2H assay showing the interaction between MIO1/SLB1 and BS1. MIO1/SLB1 interacts with BS1 through its WD40 repeat domain. AD+BD was used as the control. -2, SD/-Leu/-Trp; -4, SD/-Ade/-His/-Leu/-Trp. (D) BiFC assay showing the interaction between MIO1/SLB1 and BS1 in the nuclei of tobacco leaf epidermal cells. MIO1-nYFP and BS1-cYFP are coexpressed in leaves of tobacco, DAPI signals indicate the nuclei. (E) N-terminal WD40 repeat domain of MIO1/SLB1 (MIO1 WD40) interacts with BS1 *in vitro*. His-BS1 was pulled down (PD) by GST-MIO1 WD40 immobilized on glutathione sepharose, and analysed by immunoblotting (IB) using an anti-His antibody. GST was used as a negative control. (F) MIO1/SLB1 regulates BS1 stability *in vitro*. The His-BS1 fusion protein was detected with the His antibody. MG132 was used to inhibit the proteasome activity. The total proteins extracted from plants were used as a loading control. (G) Yeast two-hybrid (Y2H) assay showing the interaction between MIO1/SLB1 and MtKIX. -2, SD/-Leu/-Trp; -4, SD/-Ade/-His/-Leu/-Trp. (H-K) Expression of *MtCYCD3;3* (H), *MtCDKB1;1* (I), *MtE2Fb* (J), and *MtKRP3* (K) in the WT and *mio1* mutant, for which *MtActin* served as the internal control. SAM, shoot apical meristem; P, plastochron. Values indicate the mean±SD (*n*=3); asterisks indicate significant differences from the WT (*P<0.05, **P<0.01; Student’s *t*-test). (L-U) RNA *in situ* hybridization of *MtH4* (*Medicago truncatula HISTONE4*) and *MtCYCD3;3*. Longitudinal sections of shoot apices (SAM+P1-P3) and P4 of the wild type (WT) and *mio1* mutant were hybridized with anti-sense probe of *MtH4* (L to O) and *MtCYCD3;3* (P to S). The shoot apices and P4 were collected from three-week-old WT and *mio1* seedlings. P+number, plastochron; asterisks denote the shoot apical meristem. The *MtH4* and *MtCYCD3;3* sense probes were used as the control (T and U). Scale bar=50 μm.

To uncover the mechanism underlying the control of organ size by MIO1/SLB1, we performed protein-protein interaction assays to analyse the interaction between MIO1/SLB1 and other potential components of the ubiquitin-proteasome pathway. MtASK, the ortholog of ASK1/2 in *M. truncatula*, was identified by phylogenetic analysis and its interaction with MIO1/SLB1 was tested using Y2H and BiFC assays. According to these results, MIO1/SLB1 physically interacted with MtASK in both yeast and tobacco leaf cells (Fig. 6A, B). Similarly, the interaction between MIO1/SLB1 and BS1 was also verified by the Y2H and BiFC assays (Fig. 6C, D). Further analysis of the Y2H results revealed that MIO1/SLB1 is capable of interacting with BS1 through its C-terminal WD40 repeat domain (Fig. 6C), which was confirmed by the results from the *in vitro* pull-down experiment (Fig. 6E). Hence, MIO1/SLB1 could interact not only with MtASK, which is predicted to form an SCF E3 ubiquitin ligase, but also with BS1 in a way possibly related to protein degradation.

Given the similar phenotypes of *MIO1*-overexpressing and *bs1* mutant plants (Ge *et al*, 2016), we suspected that MIO1/SLB1 might regulate the stability of BS1 protein to control organ size in *M. truncatula*. To test this, total proteins were extracted from WT, *mio1* mutant, and *MIO1*-overexpressing plants, and mixed with equal amounts of *E. coli*-expressed His-BS1 fusions, followed by incubation at 4 °C with gentle shaking. Samples were then removed at different time points for a gel quantification analysis. Evidently, the amount of His-BS1 decreased after 30 min of incubation compared with what it was at the start (time 0: time point=0 min) in both the WT and *MIO1*-overexpressing plants (Fig. 6F). However, the His-BS1 protein degraded more slowly when incubated with the proteasome inhibitor MG132 (Fig. 6F), indicating that the stability of BS1 is affected by the proteasome.

Additionally, the degradation of His-BS1 fusion protein appeared to be much slower in samples incubated with total proteins from the *mio1* mutant but faster in samples incubated with proteins from the *MIO1-*overexpressing lines, when compared with the WT (Fig. 6F), indicating that the stability of BS1 protein is negatively regulated by MIO1/SLB1 *in vitro*. Taken together, these results suggested that MIO1/SLB1 might form an SCF E3 ubiquitin ligase complex with MtASK so as to modulate BS1 stability and control the lateral organ size in *M. truncatula*. MtKIX8 was identified during the Y2H library screening for potential interacting proteins of MIO1/SLB1, and was verified to physically interact with MIO1/SLB1 (Fig. 6G). Since MIO1/SLB1 remarkably influences cell proliferation and the number of cells, the expression of several representative cell-cycle genes were analysed in the *mio1* mutant and WT plants. The expression of *MtCYCD3;2*, *MtCDKB1;1*, *MtE2Fb*, and *MtKRP3* were analysed by qRT–PCR in SAM (shoot apical meristem) and P1 (plastochron 1)-P8 of six-week-old *mio1* mutant and WT plants (Supplementary Fig. S9). The expression of the predicted cell division activators *MtCYCD3;2* (Fig. 6H; Supplementary Fig. S10A), *MtCDKB1;1* (Fig. 6I; Supplementary Fig. S10B), and *MtE2Fb* (Fig. 6J; Supplementary Fig. S10C) were all decreased in the *mio1* mutant when compared with the WT, especially in the juvenile leaves. By contrast, the expression of the predicted cell division repressor *MtKRP3* (Fig. 6K; Supplementary Fig. S10D) was increased in *mio1* mutant at the P4 and P5 stages, where cell division activity is supposed to be high. We then carried out an *in-situ* hybridization assay to analyse the expression of *MtH4* (*Medicago HISTONE4*) (Fig. 6L-O) and *MtCYCD3;3* (Fig. 6P-S) at the early leaf development stages of WT and *mio1* mutant plants. These results showed that both *MtH4* and *MtCYCD3;3* were expressed in the SAM and P1 to P4 of both the WT and *mio1* mutant, but the *mio1* mutant had much weaker signals. These findings suggested that MIO1/SLB1 could influence the expression of the core cell-cycle genes; hence, it was suspected to regulate cell proliferation and lateral organ size.

### MIO1/SLB1 influences pulvinus development and leaf movement

**Fig. 7.**
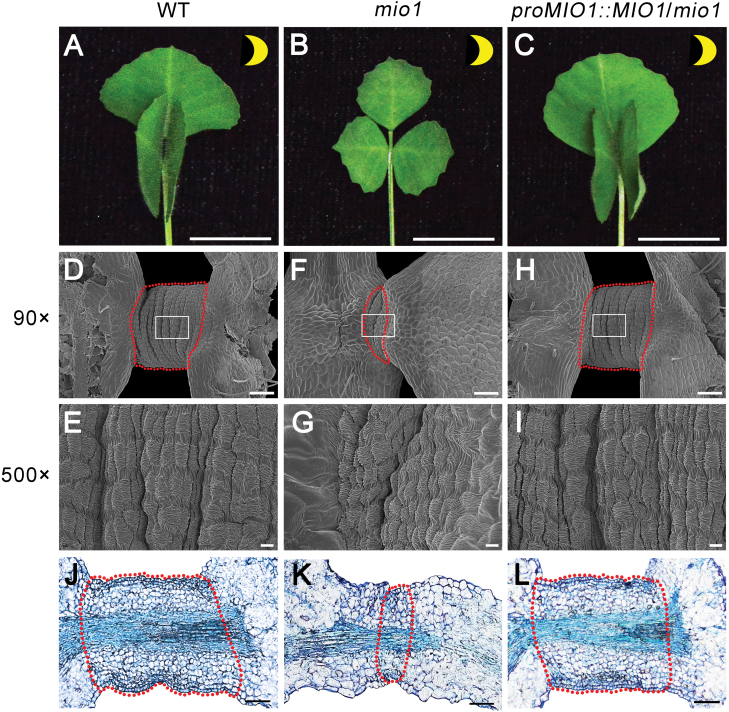
*MIO1*/*SLB1* rescues the leaf movement and pulvinus of the *mio1* mutant. (A-C) *MIO1*/*SLB1* restored the defective leaf movement of the *mio1* mutant. A representative trifoliate leaf of wild type (WT) (A), *mio1* mutant (B), and complemented *mio1* line *proMIO1::MIO1*/*mio1* (C) at night. Scale bar=1cm. (D-I) *MIO1*/*SLB1* rescued the defective pulvinus structure of the *mio1* mutant. Scanning electron microscope (SEM) images of WT (D and E), *mio1* mutant (F and G), and complemented *mio1* line (H and I). The pulvini were highlighted by the red dotted line. Open boxes in D, F, and H, indicate those areas shown in E, G, and I, respectively. Scale bar=100 μm (D, F, H),10 μm (E, G, I). (J-L) Pulvinus longitudinal-sections of the WT (J), *mio1* mutant (K), and complemented *mio1* line (L). The pulvinus region in each longitudinal section is highlighted by the red dotted line. Scale bar=10 μm.

Leaf movement is driven by a motor organ- the pulvinus - that is commonly observed in legume species (Darwin, 1880; Satter *et al.*, 1990; Koller, 2011; Mancuso and Shabala, 2015). Besides its decreased organ size, the *mio1* mutant also showed defective leaf movement when compared with the WT (Fig. 7A, B).

Generally, all the leaflets of WT engaged in reversible movement, that is, remaining in a horizontal (open) position during the day and vertical (closed) at night (Supplementary Fig. S11). In contrast to the WT, leaves of the *mio1* mutant featured a suite of defects in terms of leaf closure (Supplementary Fig. S11C, D). Generally, leaf movement was absent in the first and second trifoliate leaves (L1, L2), after which the degree of leaflet rotation gradually increased in the third to sixth trifoliate leaves (L3–L6), followed by it progressively decreasing in the seventh to tenth trifoliate leaves until it was completely lost (L7–L10; Supplementary Fig. S11A, C, D). These defects in leaf movement were rescued when the coding sequence of *MIO1*/*SLB1*, which was driven by its native promoter, was expressed in the *mio1* mutant (Fig. 7C; Supplementary Fig. S12).

To investigate the causes behind the defective leaf movement in the *mio1* mutant, we analysed the structure of its pulvinus. The pulvini of *mio1* mutants were shortened or even completely absent from the base of its leaflets, and this followed a developmental stage-dependent manner that matched the defects in leaf movement described above (Supplementary Fig. S13). The pulvinus was completely absent in the L1 and the L2 leaves, but progressively recovered from the L3 to L6, yet it gradually disappeared again from the L7 to L10 until it was lacking entirely (Supplementary Fig. S13). From the SEM observations, we found that the knitted wool-like structure of the pulvinus was partially or completely absent from the boundary region between the leaflet and petiole in the *mio1* mutant in comparison with the WT (Fig. 7D-G; Supplementary Fig. S14). This abnormal pulvinus structure was restored to a WT-like phenotype in the complemented transgenic lines (Fig. 7H, I).

Moreover, the anatomical analysis uncovered a substantially decreased number of motor cells in the typically defective pulvinus of *mio1* mutant when compared with those of the WT (Fig. 7J, K), but this was also fully rescued in the complemented plants (Fig. 7L). These results indicated that the defective pulvinus of *mio1* mutant likely arose from reduced motor cell division and final cell counts. Auxin was able to specifically accumulate in the pulvinus of WT (Zhou *et al.*, 2012; Supplementary Fig. S15A, B), but this signal disappeared in the *mio1* mutant (Supplementary Fig. S15C, D). In conclusion, we have shown that MIO1/SLB1 is also necessary for robust pulvinus development and leaf movement in *M. truncatula*.

## Discussion

### MIO1/SLB1 positively regulates primary cell proliferation to control organ size

The final organ size of multicellular plants is determined by a complex coordination of cell division and expansion (Gonzalez *et al.*, 2012). Our results demonstrate that MIO1/SLB1 acts as a positive regulator of organ size by promoting the number of cells in *M. truncatula*. The time-course analysis of epidermal cell counts in developing leaflets indicated that MIO1/SLB1 promoted cell proliferation during the early development stages (Fig. 5P); the stomatal index (a credible indicator of meristemoid cell division), epidermal cell pattern, and duration of leaf area expansion were not influenced by either loss-of-function or overexpression of *MIO1/SLB1* when compared with the WT (Fig. 5H–J, Q; Supplementary Figs S5; S7; S8). These results suggest that MIO1/SLB1 functions as a facilitator of primary cell division, rather than meristemoid cell proliferation, to modulate organ size during development.

MIO1/SLB1 and its orthologs SAP (in Arabidopsis) and LL (in cucumber) perform a conserved function - to positively regulate organ size during plant development, but interestingly, their effects on cell proliferation and cell size seem to follow different pathways. While MIO1/SLB1 positively controls primary cell division, SAP mainly acts to promote meristemoid cell proliferation during development (Wang *et al.*, 2016). This functional variation might be caused by the differences in function of their downstream regulators between the Fabaceae and Brassicaceae. Previous studies have confirmed that PPD (the substrate of SAP) and its ortholog BS1 in *M. truncatula* act as negative regulators of meristemoid proliferation and primary cell division, respectively (White, 2006; Ge *et al.*, 2016). The loss-of-function of *MIO1*/*SLB1* in *M. truncatula* and *LL* in *C. sativus* led to an increased and decreased cell size, respectively, when compared with the WT (Yang *et al.*, 2018). A probable explanation for this functional divergence in cell size is the different mutational patterns and compensatory effects of *mio1* and *ll* mutants. The *mio1* mutant arose from the *Tnt1* retrotransposon insertion, which completely eliminated the *MIO1/SLB1* transcript, so its cells may expand in an attempt to compensate for a drastic loss in cell division (Horiguchi and Tsukaya, 2011). Since the *ll* mutant arose from a single nucleotide substitution (Yang *et al.*, 2018), LL might retain some partial functioning: it might remain below the threshold to confer full function, yet still above the threshold needed to resist compensation. Irrespective of compensation and species differences, both cell proliferation and cell expansion clearly contribute to determining organ size, and they account for the conserved function of MIO1/SLB1, SAP, and LL in their native plant species.

### MIO1/SLB1 controls final organ size by regulating BS1 stability and expression of cell-cycle genes

Based on previous reports (Ge *et al.*, 2016; Wang *et al.*, 2016), we proposed that the F-box protein MIO1/SLB1 could form part of an SCF E3 ubiquitin ligase complex, to regulate organ size via targeting its potential substrate BS1 for ubiquitination and degradation in *M. truncatula*. Protein-protein interaction assays indicated that MIO1/SLB1 could directly interact with MtASK and BS1 (Fig. 6A-E). Our analysis also showed that the protein stability of BS1 could be modulated by MIO1/SLB1 with sensitivity to the inhibitor MG132 (Fig. 6F). These observations are consistent with BS1 being recruited by MIO1/SLB1 into the ubiquitin-proteasome pathway to control its stability.

Nevertheless, the His-BS1 fusion could still be degraded by proteins extracted from the *mio1* mutant, albeit at a slower rate (Fig. 6F), which suggests that additional factors may coexist with MIO1/SLB1 to regulate the abundance of BS1. It is thus conceivable that in *M. truncatula,* MIO1/SLB1 positively regulates organ size, in part, by forming the SCF E3 ubiquitin ligase to target the organ size repressor BS1 for degradation. Identifying the additional factors that co-regulate BS1 and elucidating their molecular mechanisms will enlighten our understanding of organ size regulation in legumes.

Cyclin-dependent kinases (CDKs) are the primary regulators of eukaryotic cell cycle progression, whose catalytic activity depends on the binding and activation of cyclins (CYCs; Joubès *et al.*, 2000). For example, the plant-specific CDKs are necessary for proper cell cycle progression (Andersen *et al.*, 2008; Nowack *et al.*, 2012), and CYCD3s are vital regulators that control cell division and expansion by regulating the duration of mitotic phase and the mitosis-to-endocycle transition (Dewitte *et al.*, 2007).

Kip-related proteins (KRPs), another kind of essential cell cycle regulator, which usually exhibit cyclin-dependent kinase binding specificity, are the negative regulators of CDKs (Verkest *et al.*, 2005; De Almeida Engler *et al.*, 2009). Other work has revealed that the conserved transcription factors E2Fs play crucial roles in several pathways related to plant cell division and differentiation; for instance, E2Fb is an activator of cell cycle progression in Arabidopsis (Magyar *et al.*, 2005; Sozzani *et al.*, 2006). The expression of three genes encoding predicted positive regulators of the cell cycle in *M. truncatula*, namely *MtCYCD3;2, MtCDKB1;1*, and *MtE2Fb*, were down-regulated in juvenile leaves of *mio1* mutants (Fig. 6H-J; Supplementary Fig. S10A-C). In contrast, the expression of *MtKRP3*, the predicted cell cycle repressor, was up-regulated in *mio1* mutant (Fig. 6K; Supplementary Fig. S10D). The *in-situ* hybridization assay further confirmed the disrupted expression profiles of cell cycle genes in the *mio1* mutant (Fig. 6L–U). These results suggest that MIO1/SLB1 could regulate the expression of cell cycle genes to manipulate cell proliferation and expansion in *M. truncatula*. The variation in expression profiles of cell cycle genes in the *mio1* mutant is similar to those resulting from the breakdown of *LL*, the ortholog of *MIO1*/*SLB1* in cucumber (Yang *et al.*, 2018). BS1 and its ortholog PPD also influence cell cycle gene expression in *M. truncatula* and *A. thaliana*, respectively (Gonzalez *et al.*, 2015; Ge *et al.*, 2016). So, collectively, these clues suggest that the MIO1/SLB1-related pathway that is relevant to organ size control through regulation of cell cycle gene expression during plant development, might be conserved.

*Medicago truncatula* KINASE-INDUCIBLE DOMAIN INTERACTING 8 (MtKIX8), the homolog of KIX8 in Arabidopsis, was identified during the Y2H library screening for potential interacting proteins of MIO1/SLB1, and then verified to physically interact with MIO1/SLB1 via the Y2H assay (Fig. 6G). In Arabidopsis, SAP interacts with PPD and KIXs to target the PPD-KIX complex for degradation (Wang *et al.*, 2016; Li *et al.*, 2018). Accordingly, it is likely that the putative MIO1/SLB1-BS1-MtKIX module is playing a vital role in plant organ size determination analogous to the SAP-PPD-KIX module. Yet interestingly, it is possible that the MIO1/SLB1-BS1-MtKIX module has disappeared from the Poaceae, based on our inspection of available genome sequences (Supplementary Fig. S2). Further investigation of this complex may advance our understanding of differences in molecular mechanisms of lateral organ expansion between eudicot and monocot plant species.

The organ size of legume crops and forage is one of the most important agronomic traits because it is closely related to final yield and quality. Those previously reported genes in *M. truncatula*, such as *SGL1* (*SINGLE LEAFLET1*), *PALM1*, *LLS1* (*LATERAL LEAFLET SUPPRESSION1*), and *PINNA1* (*PENTAFOLIATA1*), all provide important clues as to how the total leaf area and biomass might be promoted through manipulating the number and size of leaflets (Wang *et al.*, 2008; Chen *et al.*, 2010; He *et al.*, 2020; Zhao *et al.*, 2020). From the present study, the predicted MIO1/SLB1-BS1 module would be a useful candidate for the genetic manipulation of crop yields, via increased seed size or total biomass.

### MIO1/SLB1 influences pulvinus development in *M. truncatula*

The reversible leaf movement of legumes is driven by the pulvinus, a specialized motor organ (Satter *et al.*, 1990; Mancuso and Shabala 2015). An abnormal pulvinus initiation or development would directly influence leaf movement (Chen *et al.*, 2012; Zhou *et al.*, 2012; Gao *et al.*, 2017). Loss-of-function of *MIO1*/*SLB1* led to impaired leaf movement which resulted from the extremely shortened pulvinus in *M. truncatula* (Fig. 7B, F; Supplementary Fig. S13). However, this defect was fully rescued when the coding sequence (CDS) of *MIO1*/*SLB1* was transformed into the *mio1* mutant (Fig. 7C, H). These results provide evidence that *MIO1*/*SLB1* function is necessary to ensure the pulvinus develops normally in *M. truncatula*. Although the molecular mechanism underpinning how MIO1/SLB1 regulates pulvinus development is still unclear, there are several possibilities based on the available clues. On the one hand, MIO1/SLB1 might directly or indirectly regulate certain pulvinus identity genes, such as *ELP1* and *GmILPA1* (Chen *et al.*, 2012; Zhou *et al.*, 2012; Gao *et al.*, 2017). ELP1 is a plant-specific lateral organ boundaries domain (LBD) transcription factor that determines the pulvinus identity in legumes through its conserved function (Chen *et al.*, 2012); GmILPA1 was found to ensure the normal progression of the motor cell cycle during pulvinus development in soybean (Gao *et al.*, 2017). It would be worth analysing the genetic interaction between *MIO1*/*SLB1* and *ELP1*, or the ortholog of *GmILPA1*, during pulvinus morphogenesis. On the other hand, our anatomical examinations showed that the shortened pulvini of *mio1* mutant mainly originate from a vastly decreased number of motor cells (Fig. 7K). It seems likely that MIO1/SLB1 regulates pulvinus development through modulating the cell division of the motor organ, similar to the regulation of organ size, by taking part in the genetic pathway of cell cycle control. Loss-of-function of *MIO1*/*SLB1* led to defective leaf movement in a developmental stage-dependent manner (Supplementary Fig. S11); however, this differs from the completely lost pulvinus as found in the *elp1* mutant (Chen *et al.*, 2012). This suggests that additional extant but unknown regulators ought to function synergistically with MIO1/SLB1 to regulate pulvinus development in *M. truncatula*. Another attractive scenario is that auxin specifically accumulated in the WT pulvinus but disappeared in the *mio1* mutant (Supplementary Fig. S15). Auxin is known to have significant roles in the activation of cell cycle processes (Perrot-Rechenmann, 2010). It would be very interesting to test how MIO1/SLB1 may regulate pulvinus development through auxin-related cell proliferation processes.

## Supplementary data

The following supplementary data are available at *JXB* online.

Fig. S1. The expression of *MIO1*/*SLB1* in WT and *mio1* mutant.

Fig. S2. MIO1/SLB1 is the ortholog of LL and SAP.

Fig. S3. The distribution of F-box and WD40 repeat domain in MIO1/SLB1.

Fig. S4. The expression pattern of *MIO1*/*SLB1* in different organs of WT.

Fig. S5. Size changes of whole plants and epidermal cells in different backgrounds.

Fig. S6. The expression of *MIO1*/*SLB1* in *35S::MIO1* transgenic plants.

Fig. S7. Time-course analysis of the adaxial leaf epidermal cell pattern of WT and *mio1* mutant.

Fig. S8. Quantitative analysis of leaflet growth during different stages.

Fig. S9. Different stages of leaves were used to monitor the expression of cell-cycle genes.

Fig. S10. The expression of cell cycle genes in WT and *mio1* mutant.

Fig. S11. Loss-of-function of *MIO1*/*SLB1* results in the defect of leaf movement at different stages.

Fig. S12. Complementation of leaf movement phenotype in *mio1* mutant.

Fig. S13. Loss-of-function of *MIO1*/*SLB1* results in a series of defective pulvini.

Fig. S14. Morphological changes of epidermal cells of pulvini in *mio1* mutant.

Fig. S15. The auxin reporter *DR5rev::Green Fluorescent Protein* (*GFP*) shows the distribution of auxin in the pulvini of WT and *mio1* mutant.

Table S1. Sequence information of primers used in this study.

Table S2. Detailed information of different *mio1* mutant alleles.

erab033_suppl_Supplementaray_File001

## Data Availability

No new sequence data were published in the present paper. All sequence data included in our manuscript can be obtained from the publicly available genome of *M. truncatula* (Mt4.0v1) (http://bioinfo3.noble.org/doblast/) under the following accession numbers: MIO1 (Medtr5g097060), BS1 (Medtr1g102900), MtASK (Medtr5g022710), MtKIX8 (Medtr4g114900).
