# Supplementary material for: The F-box protein MIO1/SLB1 regulates organ size and leaf movement in Medicago truncatula
Source: J Exp Bot. 2021 Jan 28;72(8):2995–3011. doi: 10.1093/jxb/erab033 (PMC8023213; doi:10.1093/jxb/erab033)
Supplement: erab033_suppl_Supplementaray_File001 [file erab033_suppl_supplementaray_file001.pdf]

Supplemental Data:

The F-box protein MIO1/SLB1 regulates organ size and leaf movement in *Medicago truncatula*

Shaoli Zhou[1.2#], Tianquan Yang[3#], Yawen Mao[1.2], Ye Liu[1.4], Ruoruo Wang[1.2], Genwang Fangyue[1.2], Liangliang He[1.2], Baolin Zhao[1], Quanzi Bai[1.2], Shiqi Guo[1.2], Youhan Li[1], Xiaojia Zhang[1.2], Dongfa Wang[1.4], Chaoqun Wang[1.2], Qing Wu[1.2], Yuanfan Yang[1.5], Yu Liu[1], Million Tadege[6], Jianghua Chen[1*]

[1] CAS Key Laboratory of Tropical Plant Resources and Sustainable Use, CAS Center for Excellence for Molecular Plant Sciences, Xishuangbanna Tropical Botanical Garden, Chinese Academy of Sciences, Kunming, Yunnan 650223, China.

[2] University of Chinese Academy of Sciences, Beijing 100049, China.

[3] Germplasm Bank of Wild Species, Kunming Institute of Botany, Chinese Academy of Sciences, Kunming 650201, China.

[4] School of Life Sciences, University of Science and Technology of China, Hefei 230026, China.

[5] School of Ecology and Environmental Sciences, Yunnan University, Kunming 650031, China.

[6] Department of Plant and Soil Sciences, Institute for Agricultural Biosciences, Oklahoma State University, 3210 Sam Noble Parkway, Ardmore, OK 73401, USA

[#] Shaoli Zhou and Tianquan Yang contributed equally to this work.

[*] Correspondence: Jianghua Chen, Email: (jhchen@xtbg.ac.cn)

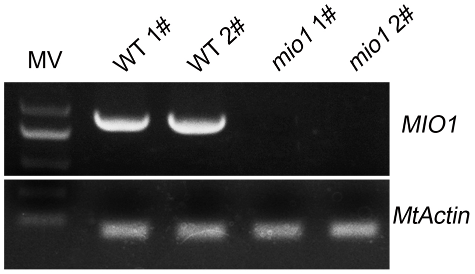

Figure. S1 The expression level of *MIO1*/*SLB1* in the WT and *mio1* mutant plants.

The total RNA was extracted from the shoot apices of 5-week-old seedlings of WT and *mio1* mutants, and the reverse transcription PCR analysis of *MIO1*/*SLB1* expression in WT and *mio1* mutant. *MtActin* was used as an internal control.

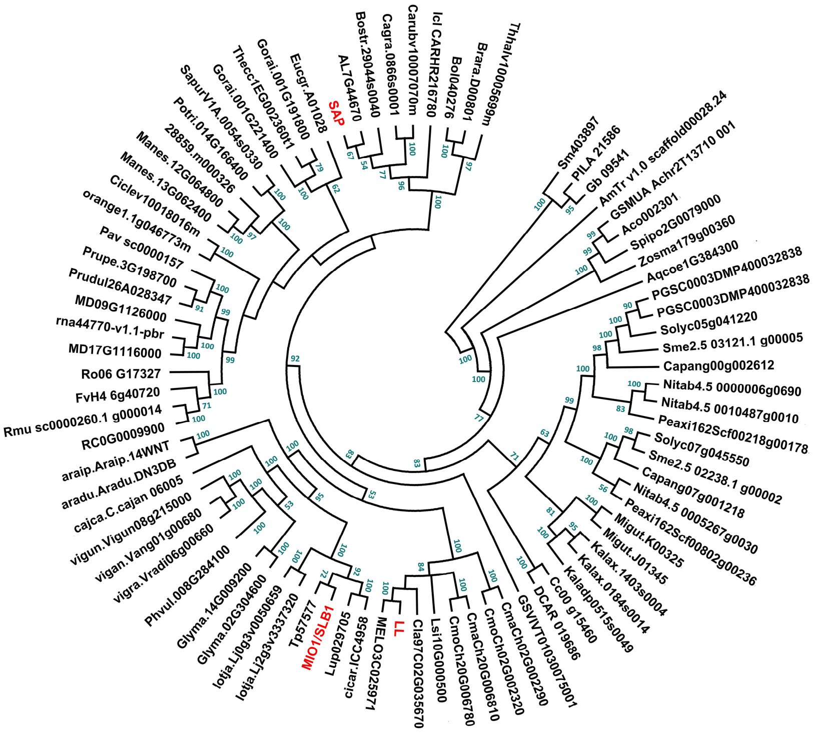

Figure. S2 MIO1/SLB1 is the ortholog of LL and SAP.

The sequences of MIO1/SLB1 homologs and protein accession numbers are showed in the figure. Based on the neighbor-Joining (NJ) criteria using MEGA5 program, bootstrap values (≥50) for each branch were indicated by numbers on lines. MIO1/SLB1 from *Medicago truncatula*, SAP from *Arabidopsis thaliana*, and LL from *Cucumis sativus* were labeled in red.

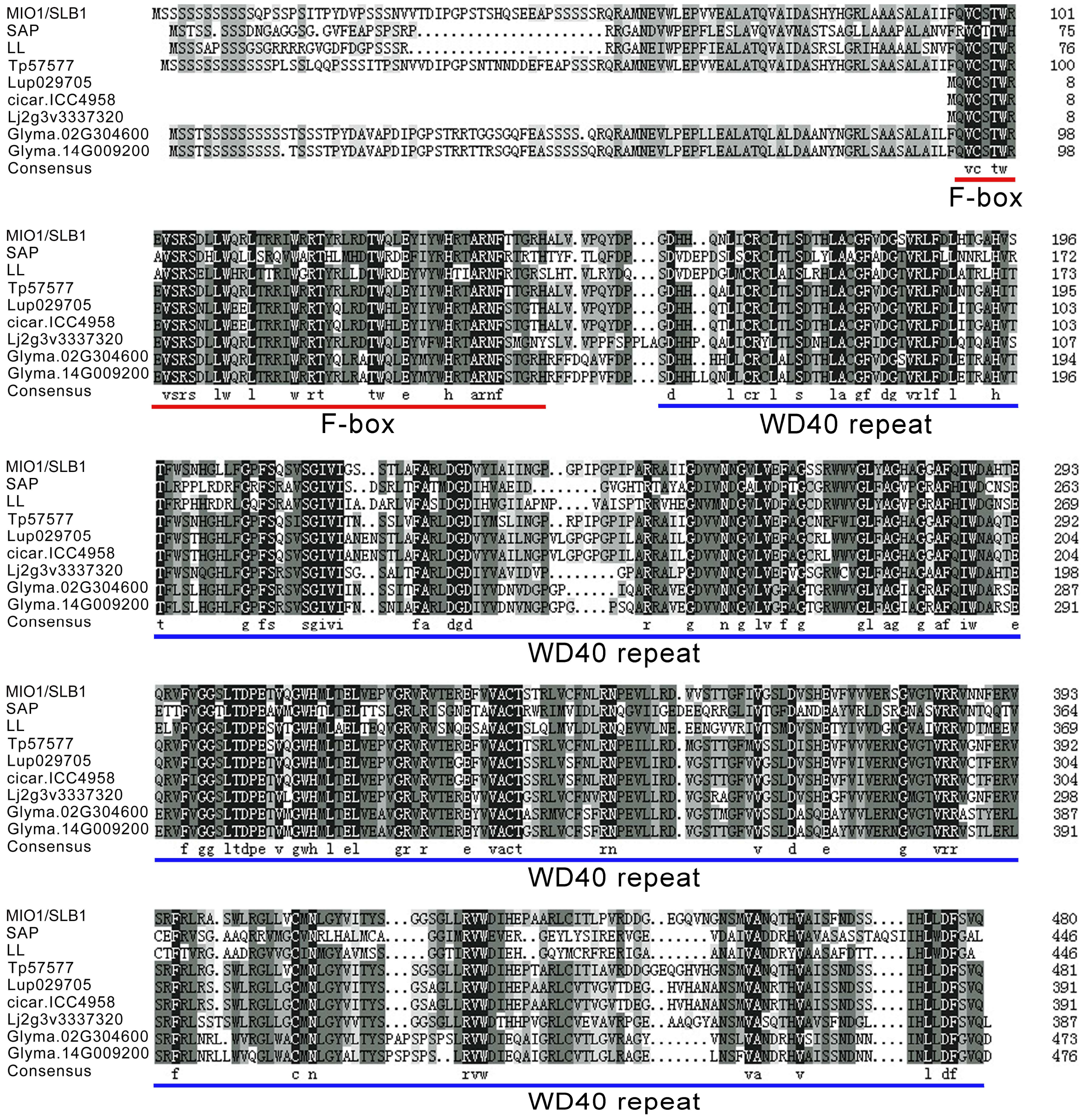

Figure. S3 The distribution of F-box and WD40 repeat domain in MIO1/SLB1 and it's homologs.

The red and blue underlines indicate the F-box and WD40 repeat domain, respectively. SAP from *Arabidopsis thaliana*, LL from *Cucumis sativus*, Tp57577 from *Trifolium pratense*, Lup029705 from *Lupinus angustifolius*, cicar.ICC4958 from *Cicer arietinum*, Lj2g3v3337320 from *Lotus japonicus*, Glyma.02G304600 and Glyma.14G009200 from *Glycine max*.

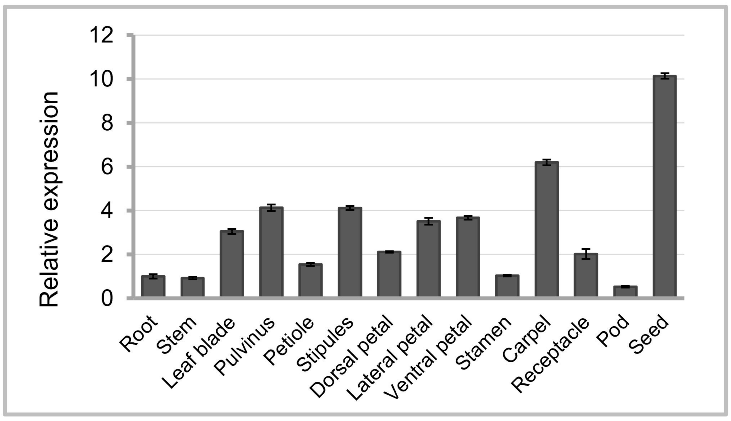

Figure. S4 The expression level of *MIO1/SLB1* in different organs of WT plants.

The *MIO1/SLB1* transcript abundance in different tissues was analyzed by qRT-PCR, *MtGAPDH* was used as an internal control. Values indicate the mean ± SD (n = 3).

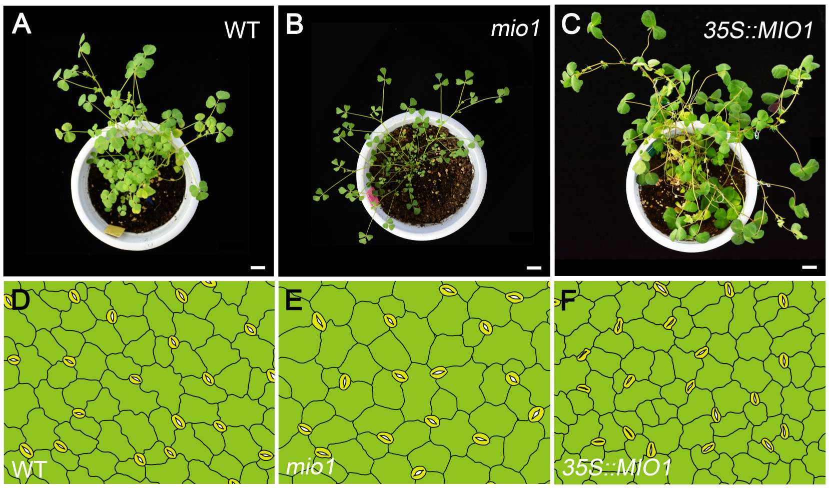

Figure. S5 Size changes of whole plants and epidermal cells in different backgrounds.

(A-C) 5-week-old seedlings of the WT **(A)**, *mio1* mutant **(B)**, and *MIO1*-overexpressing line (*35S::MIO1*) **(C)**. **(D-F)** The epidermal cell outlines of abaxial epidermis of the mature leaflet from the WT **(D)**, *mio1* mutant **(E)**, and *MIO1*-overexpressing plants **(F)**. The graphics were outlined with Photoshop software according to the photos that captured by the microscope observation, green-colored and yellow-colored cells represent epidermal cells and guard cells, respectively. Scale bar = 2cm.

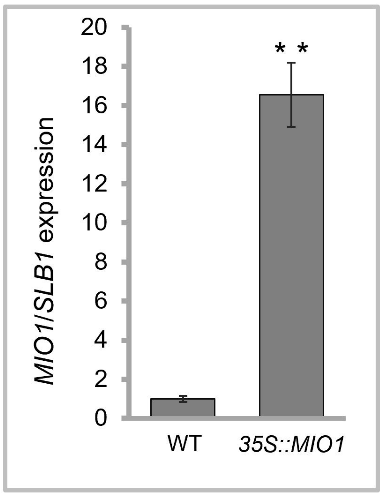

Figure. S6 The expression level of *MIO1*/*SLB1* in *35S::MIO1* transgenic plants.

The *MIO1*/*SLB1* transcript abundance of WT and *MIO1*-overexpressing lines was analyzed by qRT-PCR, *MtGAPDH* was used as an internal control. Values indicate the mean ± SD (n = 3); asterisks indicate significant differences compared with the WT (**P< 0.01; Student's t-test).

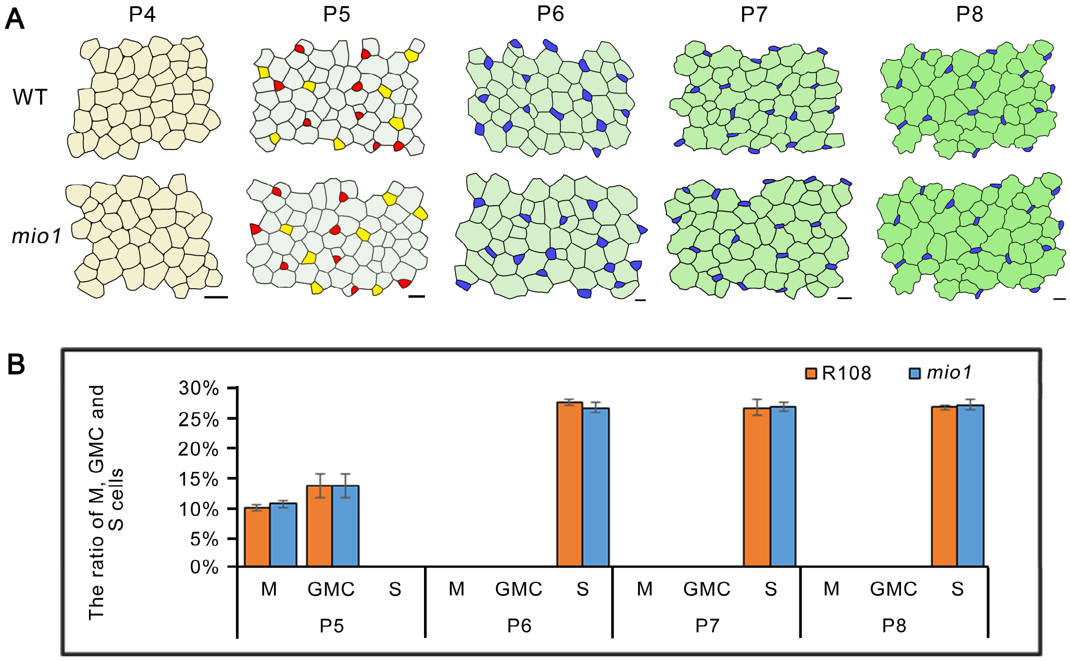

Figure. S7 Time-course analysis of the adaxial leaf epidermal cell patterns of the WT and *mio1* mutant plants.

(A) The adaxial leaf epidermal cell pattern of WT (upper) and *mio1* mutant (bottom) from the P4 (plastochron 4) to P8 which captured by the SEM (scanning electron microscope). The graphics were outlined with Photoshop software based on the photos of SEM. The meristemoid (M), guard mother cell (GMC), stomatal guard cells (S), and epidermal cells

were colored with a series of gradients of yellow, red, blue, and green, respectively.

(B) The comparison of the ratio of M, GMC, and S among adaxial leaf epidermal cell from P5 to P8 of the WT and *mio1* mutant plants. Values indicate the means ± SD (n =3 biological replicates, with te10 plants per replicate).

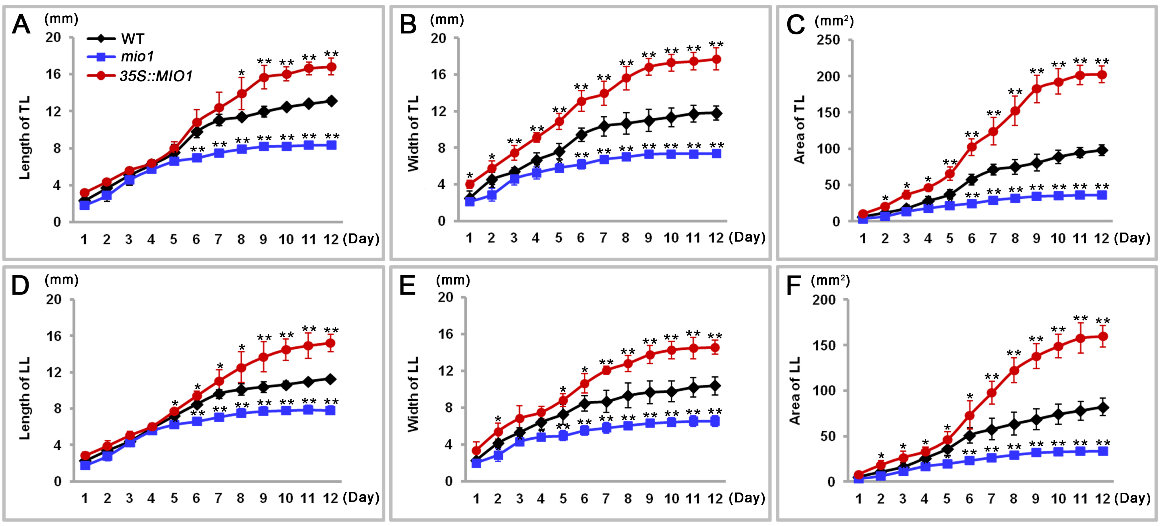

Figure. S8 Quantitative analysis of the leaflet growth during different stages.

(A-C) Growth changes in length **(A)**, width **(B)**, and area **(C)** of the terminal leaflet (TL) of WT, *mio1* mutant, and *MIO1*-overexpressing pants. **(D-F)** Growth changes in length **(D)**, width **(E)**, and area **(F)** of the lateral leaflet (LL) of WT, *mio1* mutant, and *MIO1*-overexpressing plants.

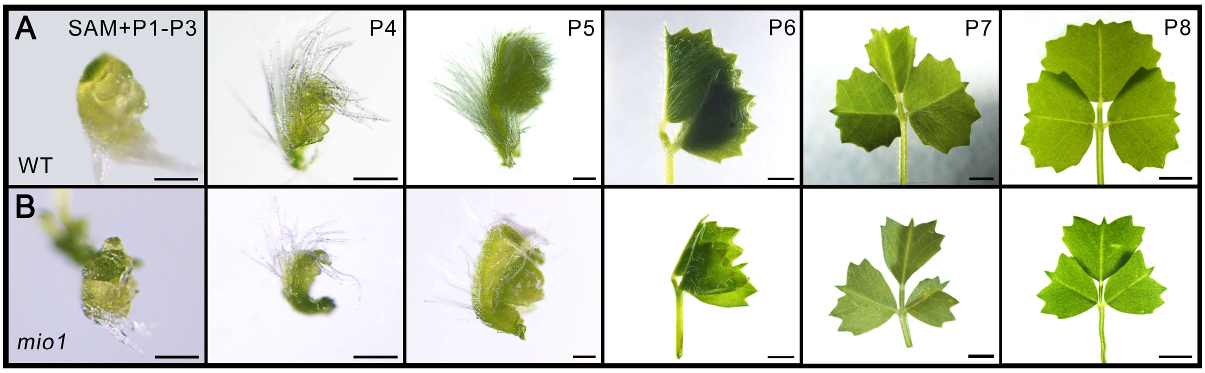

Figure. S9 The leaves of different developmental stages were used to monitor the expression level of cell-cycle genes.

Shoot apices carrying the P1 to P3 (SAM + plastochron 1 to plastochron 3), and P4 to P8 (plastochron 4 to plastochron 8) of the WT **(A)** and *mio1* mutant plants **(B)** were used to detect the expression of cell-cycle genes. Scale bar = 100 μm for SAM+P1-P3, 200 μm for P4, 500 μm for P5, 0.1 cm for P6, 0.2 cm for P7, and 0.5 cm for P8.

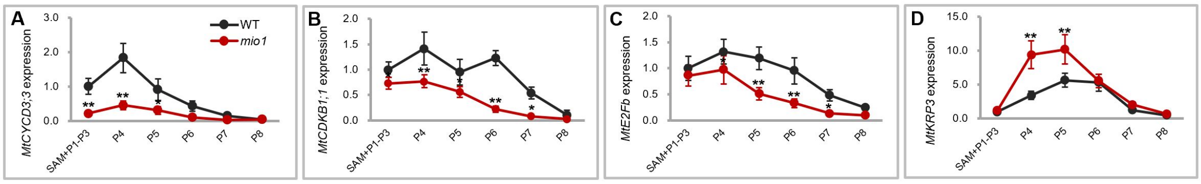

Figure. S10 The expression level of cell-cycle genes in the WT and *mio1* mutant plants.

The expression levels of the *MtCYCD3;3* **(A)**, *MtCDKB1;1* **(B)**, *MtE2Fb* **(C)**, and *MtKRP3* **(D)** in WT and *mio1* mutant plants, *MtGAPDH* was used as an internal control. SAM, shoot apical meristem; P, plastochron. Values indicate the mean ± SD (n = 3); asterisks indicate significant differences (*P < 0.05, **P < 0.01, Student's *t*-test).

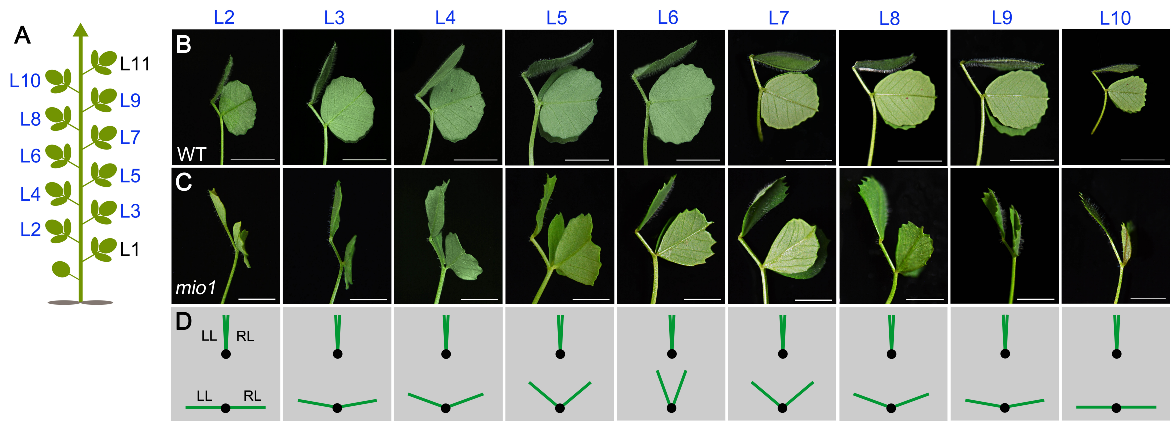

Figure. S11 Loss of function of *MIO1/SLB1* results in the defect of leaf movement at different developmental stages.

(A) The schematic of location of leaves. **(B, C)** The leaves movement of the WT **(B)** and *mio1* mutant plants **(C)** at night. L2 to L10 represent the second to tenth trifoliate leaves from base to tip. **(D)** The schematics of leaf angle of WT and *mio1* mutant plants. Black dots indicate the petioles, and the green lines represent lateral leaflets. Scale bar = 1 cm.

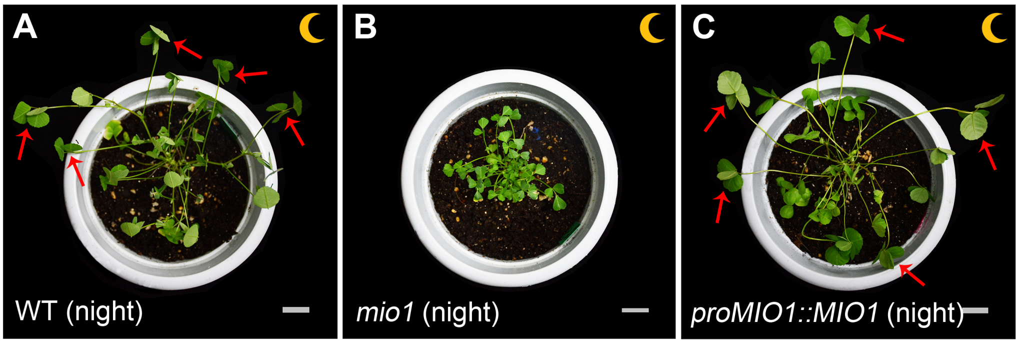

Figure. S12 Complementation of the defective leaf movement phenotype of *mio1* mutant.

The 5-week-old seedlings of the WT **(A)**, *mio1* mutant **(B)**, and complemented *mio1* mutant line **(C)** at night. The red arrows point to the closed leaves. Scale bar, 2cm.

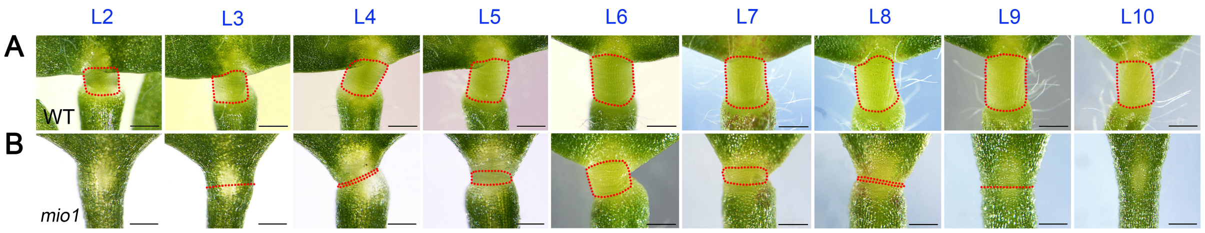

Figure. S13 Loss of function of *MIO1*/*SLB1* results in a series of defective pulvini.

The terminal leaflet pulvini (TLP) of WT **(A)** and *mio1* mutant plants **(B)**. L2 to L10 represent the second to tenth trifoliate leaves. Pulvinus region were highlighted by red dotted line. Scale bar, 1 mm.

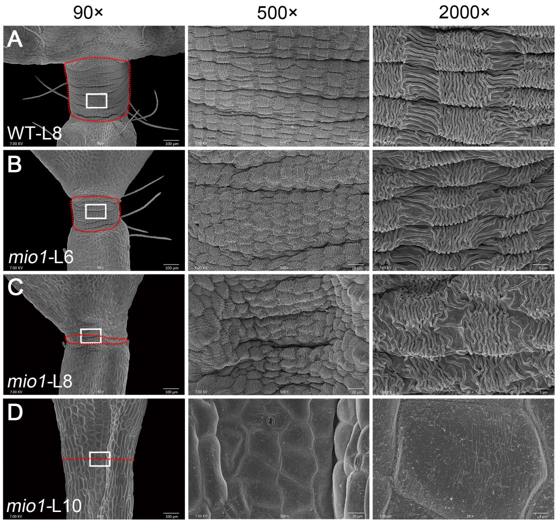

Figure. S14 Morphological changes of epidermal cells of pulvini in *mio1* mutant.

Scanning electron microscope (SEM) images of terminal leaflet pulvini (TLP) with different magnifications. The TLP of the sixth trifoliate leaf of the WT (WT-L6) plant **(A)**; the TLP of the sixth, eighth, and tenth trifoliate leaf of the *mio1* mutant (*mio1*-L6, *mio1*-L8, and *mio1*-L10) plant **(B-D)**. Pulvinus region were highlighted by red dotted line in 90×. The open boxes in 90× indicate areas shown in 500×. Scale bar = 10 μm.

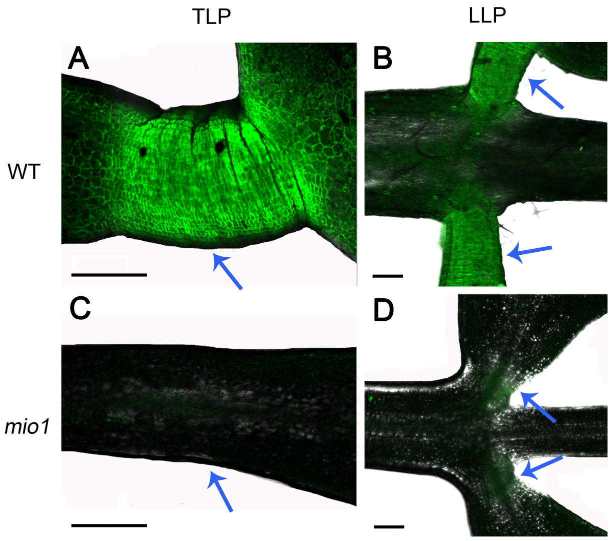

Figure. S15 The auxin reporter *DR5rev::Green Fluorescent Protein* (*GFP*) to show the distribution of auxin in the pulvini of WT and *mio1* mutant.

(A, B) *DR5rev::green fluorescent protein* (*GFP*) expression in the terminal **(A)** and lateral leaflet pulvini **(B)** of WT. **(C, D)** *DR5rev::green fluorescent protein* (*GFP*) expression in the terminal **(C)** and lateral leaflet pulvini **(D)** of *mio1* mutant. The blue arrows point to pulvini. Scale bar, 100 µm.

Table. S1 The detail information of different *mio1* mutant alleles.

**Lines number**	**Name**	***Tnt1* insertion site (bp)**	***Tnt1* inserts orientation**	**Forward/ Reverse screen**
NF3315	*mio1-1*	3780-3781	Reverse	Forward
NF8518	*mio1-2*	4209-4210	Forward	Forward
NF11386	*mio1-3*	4385-4386	Forward	Forward
NF19156	*mio1-4*	3790-3791	Forward	Reverse
NF18765	*mio1-5*	3835-3836	Forward	Reverse
NF2748	*mio1-6*	4246-4247	Reverse	Reverse

Table. S2 The sequence information of Primers used in this study.

Primer Name	Sequence	Usage
MIO1-F	TTGACCATCTCATCCAACCA	*mio1* mutant genotyping primer
MIO1-R	ATCATCTCTAACTGGGAGCG	*mio1* mutant genotyping primer

GFP-F	ACCATCTTCTTCAAGGACGACGG	*35S::MIO1-GFP* transgenic lines genotyping primer
GFP-R	ACGAACTCCAGCAGGACCATGTG	*35S::MIO1-GFP* transgenic lines genotyping primer
GUS-R	ATTGACCCACACTTTGCCGTAATGAG	*proMIO1::GUS* transgenic lines genotyping primer
proMIO1-F	GAGGTTAATTCACGCTCGAC	Amplification *MIO1* promotor primer
proMIO1-R	TAGAAACGGGACCAGAAAGG	Amplification *MIO1* promotor primer
clone c*BS1*-F	CGGAATCGGAAACGATGAA	Amplification *BS1* CDS primer
clone c*BS1*-R	GCACAATCTATCTAGTCTCAGG	Amplification *BS1* CDS primer
clone c*MtASK*-F	CACGATCACGATGTCTTCAA	Amplification *MtASK* CDS primer
clone c*MtASK*-R	GAGCATCATTCAAATGCCCA	Amplification *MtASK* CDS primer
RT-*MIO1*-F	GTATGGCTTGAACCTGTTGT	RT-PCR primer
RT-*MIO1*-R	AACAATGAATCCTGTCGTGG	RT-PCR primer
qRT-PCR *MIO1*-F	CTCCTTCATCTTCTTCCTCTCG	qRT-PCR primer
qRT-PCR *MIO1*-R	TGACACTTCCCTCCATGTTG	qRT-PCR primer
qRT-PCR *BS1*-F	AGTCCTCTTCCCTACTGACAC	qRT-PCR primer
qRT-PCR *BS1*-R	GACACTTTTCTGCTTGCCTG	qRT-PCR primer
qRT-PCR-*MtCYCD3;3*-F	GAGTTTATGTGGCGTTGTGAG	qRT-PCR primer
qRT-PCR-*MtCYCD3;3*-R	AGCACTGATTCACTTGTTCCTC	qRT-PCR primer
qRT-PCR-*MtCDKB1;1*-F	GCTGATTTAGGGCTTGGAAGAG	qRT-PCR primer
qRT-PCR-*MtCDKB1;1*-R	ATTGCTGATCCGTTGGAGTC	qRT-PCR primer
qRT-PCR-*MtKRP3*-F	AGAAGCACTAGAGAAAGCACAC	qRT-PCR primer
qRT-PCR-*MtKRP3*-R	GGACCCATTCGTACCGTC	qRT-PCR primer
qRT-PCR-*MtE2Fb*-F	AACGTCAACTCCCTTTCTCC	qRT-PCR primer
qRT-PCR-*MtE2Fb*-R	CCCGCCTTTCCAGATACAG	qRT-PCR primer
qRT-PCR-*MtActin*-F	TCAATGTGCCTGCCATGTATGT	qRT-PCR primer
qRT-PCR-*MtActin*-R	ACTCACACCGTCACCAGAATCC	qRT-PCR primer
qRT-PCR-*MtGAPDH*-F	GACTTTATTGGTGATACCAGGTCG	qRT-PCR primer
qRT-PCR-*MtGAPDH*-R	GGTCAACCACACGGGTACTGTAA	qRT-PCR primer
proMIO1::MIO1-F	ctccacttctctcactcctcATGTCTTCTTCTTCCTCCTCCT	Amplification *MIO1* CDS for construct

		primer
proMIO1::MIO1-R	gggaaattcgagctggtcacTTACTGTACACTAAAATCC	Amplification *MIO1* CDS for construct primer
35S::MIO1-F	gaacacgggggactcttgacATGTCTTCTTCTTCCTCCTCCT	Amplification *MIO1* CDS for construct primer
35S::MIO1-R	gggaaattcgagctggtcacTTACTGTACACTAAAATCC	Amplification *MIO1* CDS for construct primer
proMIO::MIO1-GFP-F	ttcatttggagaggacacgcATGTCTTCTTCTTCCTCCTCCT	Amplification *MIO1* CDS for construct primer
proMIO::MIO1-GFP-R	gttcttctcccttacccatgTTACTGTACACTAAAATCC	Amplification *MIO1* CDS for construct primer
Y2H-MIO1 F-box-F1	ggacctgcatatgGGATCCATGTCTTCTTCTTCCTCCTC	Y2H construct primer
Y2H-MIO1 F-box-R1	gatattattgacacgCCCGGGATGTCTACCTGTGGTAAAGT	Y2H construct primer
Y2H-MIO1 WD40-F1	ggacctgcatatgGGATCCGCTTTAGTGGTCCCACAATA	Y2H construct primer
Y2H-MIO1 WD40-R1	gatattattgacacgCCCGGGCTGTACACTAAAATCCAATA	Y2H construct primer
Y2H-*MtKIX1*-F	gattacgctcatatgGGATCCATGCCGCGGCCAGGGCCAAG	Y2H construct primer
Y2H-*MtKIX1*-R	gatattattgacacgCCCGGGCTACGAACCTGGCCTACCAG	Y2H construct primer
MIO1-YN-F	catttaaatctcgagGGATCCATGTCTTCTTCTTCCTCCTC	BiFC construct primer
MIO1-YN-R	ggtggcgatggatctTCTAGACTGTACACTAAAATCCAATAAAT	BiFC construct primer
BS1-YC-F	gaggaggacctgcttTCTAGAATGAACGGCGGAAGCACCG	BiFC construct primer
BS1-YC-R	cgccggacgggtaccGGATCCGCATTCTTGAACATCTTTATCATTC	BiFC construct primer
MtASK-YC-F	gaggaggacctgcttTCTAGAATGTCTTCAACAAGAAAGATCAC	BiFC construct primer
MtASK-YC-R	cgccggacgggtaccGGATCCTTCAAATGCCCATTGGTTTTCC	BiFC construct primer
SALK088833-LP	AAATAGGATTTGCCATTCCATC	*sod3-3* mutant genotyping
SALK088833-RP	ATGTGTTCTTGCCCAAACTTG	*sod3-3* mutant genotyping
His-BS1-F	cagcaaatgggtcgcGGATCC ATGAACGGCGGAAGCACCG	Protein expression construct primer
His-BS1-R	caagcttgtcgacgGAGCTCGCATTCTTGAACATCTTTATC	Protein expression construct primer
GST-MIO1WD40-F	gatctggttccgcgtGGATCCATGGCTTTAGTGGTCCCACA	Protein expression construct primer
GST-MIO1WD40-R	ctcgagtcgacccggGAATTCTTACTGTACACTAAAATCCA	Protein expression construct primer
in-situ-*MIO1*-F	ATGTCTTCTTCTTCCTCCTC	*MIO1* RNA in-situ hybridization probe amplification primer
in-situ-*MIO1*-R	TGTAATACGACTCACTATAGGGCCTGTACACTAAAATCCAATA	*MIO1* RNA in-situ hybridization probe amplification primer
